# Heat alerts and emergency department utilization: evidence from San Diego County, California

**DOI:** 10.1186/s12940-026-01325-4

**Published:** 2026-07-28

**Authors:** Leire Luque-García, Oghenetanure R. Enaworu, William Bruno, Edward Castillo, Theodore C. Chan, Wael K. Al-Delaimy

**Affiliations:** 1 Herbert Wertheim School of Public Health and Human Longevity Science, University of California San Diego, La Jolla, CA 92093 USA; 2 Chao Family Comprehensive Cancer Center, University of California Irvine, Irvine, CA 92868 USA; 3 Department of Emergency Medicine, University of California San Diego, La Jolla, CA 92093 USA

**Keywords:** Extreme heat, Early warning system, Heat alerts, Heat warnings, Heat advisories, Emergency department visits

## Abstract

**Background:**

Extreme heat poses a significant risk to human health, underscoring the importance of early warning systems in mitigating such impacts. However, evidence regarding their effectiveness remains limited, particularly for emergency department (ED) visits. This study aimed to assess the associations between heat advisory and warning issuance and performance and ED visits in San Diego County, California, and to further examine differences by cause and demographic characteristics.

**Methods:**

Daily adult ED visit data for the warm season (May–October) were linked to historical heat alerts and high-resolution gridded temperature data. ED visits were modeled using Poisson regression with ZIP code fixed effects, controlling for temperature, seasonality, and temporal factors. Main analyses evaluated associations between alert issuance and ED visits across 2012–2023, while secondary analyses examined differences by alert performance, classified as true positive, false positive, false negative, or true negative based on temperature-based criteria in effect during 2012–2016.

**Results:**

Heat advisories were associated with a 2.1% increase in all-cause ED visits (95% CI: 0.4, 3.9), as well as larger increases in neurological (13.5%; 95% CI: 1.3, 27.1) and heat-related visits (21.8%; 95% CI: 1.6, 46.1), while warnings were exclusively associated with increased heat-related ED visits (35.8%; 95% CI: 3.5, 78.3). In subgroup analyses, associations with all-cause ED visits were more pronounced among females and older adults, with warnings associated with a 12.7% increase among those aged 65 years and older (95% CI: 6.8, 18.8). During 2012–2016, heat alerts exhibited high specificity but limited sensitivity, and their performance had minimal overall influence on ED visits. Results were robust across alternative model specifications.

**Conclusion:**

Heat advisories and warnings were associated with limited changes in ED utilization, primarily confined to heat-related conditions and to females and older adults.

**Supplementary Information:**

The online version contains supplementary material available at https://doi.org/10.1186/s12940-026-01325-4.

## Introduction

Exposure to extreme heat has been associated with adverse health outcomes, including increased morbidity and mortality [1]. Importantly, while the association between heat and mortality has been well characterized [2, 3], the effects of extreme heat on morbidity, particularly in relation to emergency department (ED) utilization, remain comparatively understudied, despite ED visits constituting a sensitive indicator of heat-related health impacts, reflecting early physiological stress and preceding more severe outcomes [4]. In this regard, recent evidence from California demonstrates that temperature extremes affect morbidity and mortality differently. Specifically, while mortality and hospitalizations increased during both heat and cold extremes, ED visits increased linearly with rising temperatures [5], suggesting that the morbidity burden associated with heat is not limited to formally defined extreme conditions.

The physiological limits of heat tolerance are inherently finite and are influenced by internal and external factors that determine susceptibility and vulnerability, respectively. Importantly, deviations from historical temperature norms can impair both heat tolerance and the capacity for physiological adaptation, particularly among older adults, individuals with chronic conditions, those exposed to sustained occupational heat, and populations experiencing socioeconomic disadvantage [1, 6].

In response to the health risks posed by extreme heat events—particularly among highly susceptible and vulnerable populations—public health authorities, health departments, and other government agencies in the United States have implemented a range of strategies to protect the public from heat-related adverse health effects. Among these strategies, Heat Response Plans (HRPs) constitute coordinated frameworks that describe and organize activities aimed at preventing heat-related morbidity and mortality during periods of extreme heat [7]. A core component of HRP is the use of early warning systems to support timely and targeted public health action and risk notification. These warning systems typically integrate meteorological forecasts to generate graded heat alerts that are issued to trigger timely public health interventions intended to reduce adverse health impacts during periods of extreme heat [8].

San Diego County exemplifies this approach through the implementation of a comprehensive Excessive Heat Response Plan (EHRP) [9], organized into three activation phases: Phase I (Seasonal Readiness), Phase II (Advisories), and Phase III (Warnings). Phase I emphasizes preparedness and public awareness, while Phases II and III are activated in response to heat advisories and warnings issued by the National Weather Service (NWS), collectively referred to as heat alerts, which are typically issued 12 to 48 h in advance of onset [10]. Upon activation of Phases II or III, operational protocols are triggered for hospitals and public health agencies, including enhanced surveillance, activation of cooling resources, and targeted outreach to high-risk populations. Heat alert criteria in San Diego County have evolved over time. Until 2016, alerts were issued based on forecasted maximum temperatures, with the NWS applying zone-specific and month-specific temperature thresholds across the county’s four climatic zones. In 2017, the NWS adopted the HeatRisk framework, which integrates forecasted temperatures into a climatological context, accounting for temperature anomaly, overnight heat retention, event duration, and seasonal acclimatization to generate a standardized five-level color-coded risk index [11]. As part of this transition, heat advisories in desert zones were discontinued.

However, heat alerts may not always perform as expected, as changes in alert criteria over time and inherent uncertainty in weather forecasts mean that days with comparable observed heat conditions may differ in alert status, resulting in false and missed heat alerts. This may influence risk perception and, in turn, behavioral adaptation, with subsequent implications for healthcare utilization. Specifically, false alerts, defined as days when an alert is issued but forecast heat does not materialize, may prompt protective behavioral responses that reduce ED utilization. Conversely, missed alerts, defined as days when hazardous heat occurs in the absence of a prior alert, may result in inadequate population-level warning, potentially increasing heat-related morbidity and healthcare utilization. Evaluating alert performance, beyond alert issuance alone, is therefore essential to determine whether early warning systems reduce heat-related health impacts independently of the underlying heat conditions, and to inform evidence-based improvements to their design and implementation.

Despite their public health importance, the extent to which heat alerts reduce adverse health outcomes associated with extreme heat remains unclear. Several studies have examined the effects of heat alerts on mortality [12, 13]. However, mortality reflects only the most severe end of the heat-related health impact spectrum, and morbidity outcomes, including ED visits and hospitalizations, may reflect a broader and more immediate public health burden that is not fully characterized by mortality alone. Morbidity findings across studies remain inconsistent, with many studies examining outcomes among older adults, thereby limiting the generalizability of findings to the broader population [12]. Moreover, evidence on the effects of heat alerts on morbidity outcomes by cause and demographic characteristics remains limited, despite its importance for identifying vulnerable populations to inform targeted public health interventions.

To address this research gap, we aimed to evaluate the association between NWS heat alerts and morbidity in San Diego County. We examined whether the association between heat advisory and warning issuance and ED visits varied by cause and further assessed whether the association for all-cause ED visits varied across demographic subgroups defined by biological sex, age, and ethnicity. Additionally, we examined the associations between heat advisory and warning performance, including missed and false heat alerts, and patterns of ED utilization across demographic subgroups.

## Methodology

### Study area

The study was conducted in San Diego County, California, a large and diverse region spanning more than 4,300 square miles along the border between the United States and Mexico and the Pacific coastline. The county is the second most populous in California and one of the largest in the United States, with a population exceeding three million residents. Demographically, San Diego County is highly diverse, with a large immigrant community, a substantial Hispanic population, and growing proportions of older adults and individuals with disabilities. San Diego County spans a wide range of geographic features that influence spatial variation in temperature, humidity, and precipitation across the region. As a result, the county encompasses four climatically distinct zones: Coastal, Inland, Mountains, and Deserts [14]. A detailed description of each climatic zone is provided in Supplementary Text S1.

### Emergency department data

We obtained daily ED visit data from UC San Diego Health for the years 2012 to 2023. Data from 2012 represent a partial year, as the electronic health record system was implemented in June. UC San Diego Health is the only academic health system in San Diego County and includes two major EDs (approximate annual census 90,000), located at Jacobs Medical Center in La Jolla and UC San Diego Medical Center in Hillcrest. Since our analyses were focused on the warm season, we restricted the dataset to ED visits occurring between May and October. We further restricted the study population to individuals aged 18 years and older. While pediatric heat vulnerability is recognized, the limited number of ED visits among individuals aged 0–17 years did not support robust analyses.

For each ED visit, the dataset included the date of the visit, International Classification of Diseases, Ninth Revision (ICD-9) and Tenth Revision (ICD-10) diagnosis codes, patient biological sex, ethnicity, age, and the community and ZIP code of residence. To define demographic subgroups, all-cause ED visits were stratified by sex (male or female), ethnicity (Hispanic or non-Hispanic), and age group (18–64 or 65 + years). Cause-specific ED visits were constructed using ICD-9 and ICD-10 diagnosis codes recorded for each visit. We included the following cause-specific categories: circulatory, respiratory, mental health, neurological, digestive, endocrine, urinary, accidents, and heat-related conditions. Detailed diagnostic coding for each cause-specific category is provided in Supplementary Table S1.

### Meteorological data

We obtained daily maximum, mean, and minimum temperature (°C) and relative humidity (%) from the Gridded Surface Meteorological (gridMET) dataset, a high-resolution (4 km × 4 km) product providing surface meteorological variables across the contiguous United States [15]. We initially extracted climatic variables at the ZIP code level across San Diego County and subsequently population-weighted them using constrained WorldPop data. WorldPop employs Random Forests-based dasymetric redistribution of census totals to 100 m resolution, integrating satellite-derived settlement patterns with official demographics to generate spatially explicit population distributions for precise exposure estimation [16].

### Heat alert data

We retrieved information on historical heat alerts for the years 2012–2023 from the Iowa Environmental Mesonet database, which archives official NWS heat alerts. Each record was classified as a discrete event with defined start and end dates. These heat alerts correspond to instances in which the NWS issued either an advisory or the more severe heat warning, based on forecasted conditions expected to pose health risks.

**Fig. 1:**
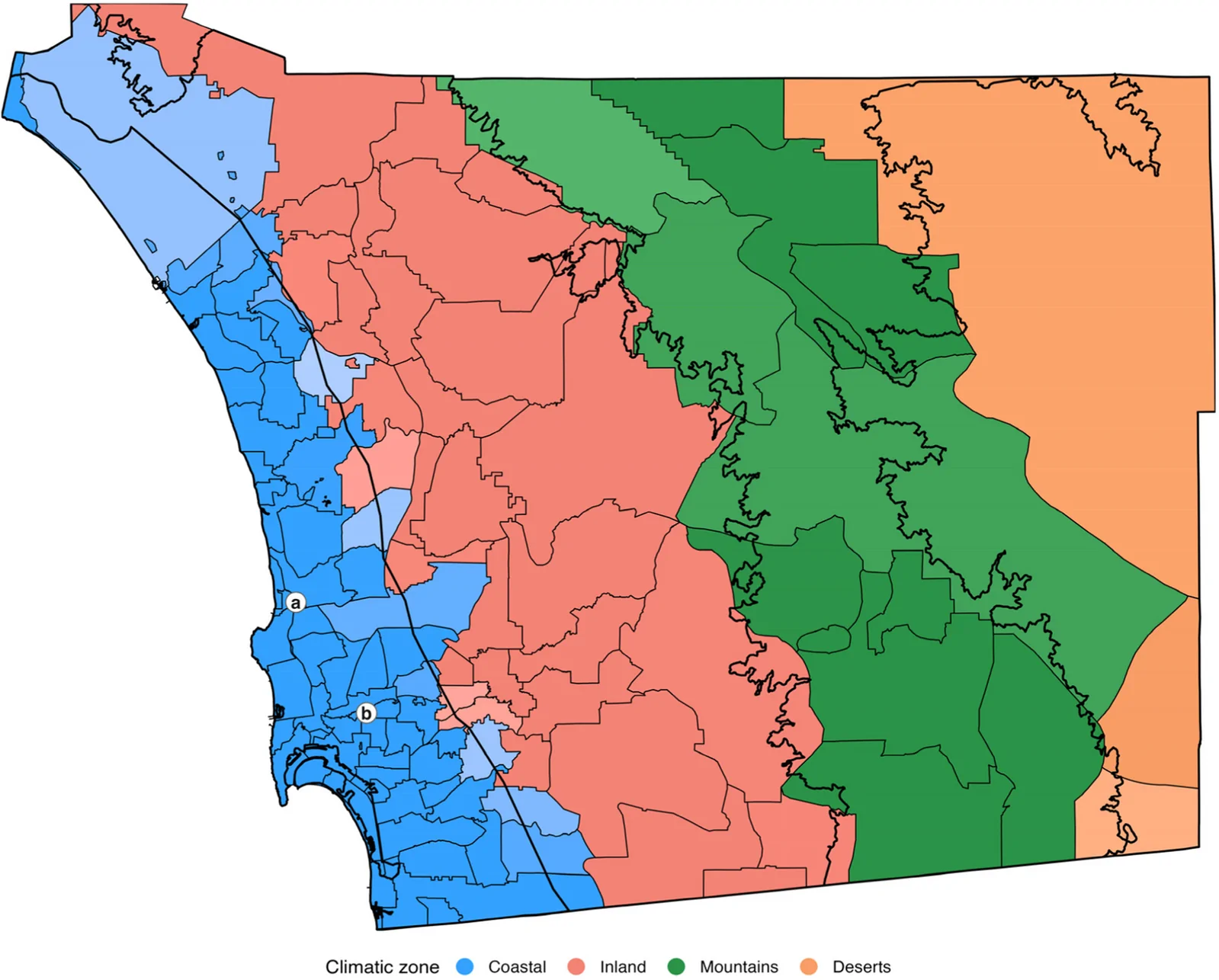
Geographic assignment of ZIP codes to climatic zones based on population distribution. Darker gradient shading represents a higher population share within the assigned climatic zone. *Note*: (a) Jacobs Medical Center in La Jolla, (b) UC San Diego Medical Center in Hillcrest

Since heat alerts are issued by climatic zone and ZIP codes constitute the geographic unit of analysis, each ZIP code in San Diego County was assigned to one of four climate zones. Assignment was based on population distribution estimated using constrained WorldPop data at 100 m resolution. For each intersection between a ZIP code and a climatic zone, the proportion of the ZIP code’s total population residing within each zone was estimated. ZIP codes were subsequently assigned to the climatic zone containing the largest share of its total population (Fig. 1).

Alert performance was evaluated over 2012–2016, the period during which heat advisories and warnings were issued under consistent, temperature-threshold-based criteria prior to adoption of the HeatRisk framework. For each ZIP code and day, the alignment between issued alerts and observed maximum temperatures was assessed by applying climatic zone-specific and month-specific thresholds (Supplementary Table S2). Each ZIP code-day observation was then classified into one of four categories:

- True Positive (TP): alert issued and threshold exceeded.
- False Positive (FP): alert issued and threshold not exceeded.
- False Negative (FN): no alert issued and threshold exceeded.
- True Negative (TN): no alert issued and threshold not exceeded.

### Statistical analyses

Descriptive statistics were computed for all exposure and outcome variables. Meteorological variables were summarized by climatic zone, both overall and stratified by alert issuance category. ED visit counts were described by demographic characteristics and cause-specific categories using frequencies and percentages. The diagnostic performance of heat alerts was then assessed across the four climatic zones using accuracy, sensitivity, specificity, positive predictive value (PPV), and negative predictive value (NPV) [17]. Definitions and corresponding formulas for each metric are provided in Supplementary Text S2.

A longitudinal ecological panel design was employed, with daily ED visit counts modeled at the ZIP code level using Poisson regression and ZIP code fixed effects. ZIP code fixed effects accounted for time-invariant spatial confounding, while time-varying confounders were addressed through explicit covariate adjustment. ZIP codes represented the most precise geographic unit available for the health data, and their boundaries remained largely stable throughout the study period, minimizing concerns about temporal misclassification. For ED visits associated with ZIP codes outside San Diego County, population-weighted meteorological exposures and alert data from the Coastal region were assigned, reflecting the coastal location of the EDs.

Main analyses assessed the association between alert issuance and daily ED visits over 2012–2023. Heat advisories and warnings were coded as separate binary indicators and modeled independently, allowing examination of differential associations by alert type. Main analyses were conducted for both all-cause and cause-specific ED visit counts. For all-cause ED visits, associations were examined separately by sex, ethnicity, and age group by including an interaction term between the alert variable and each demographic indicator. Secondary analyses examined whether all-cause ED visits differed according to alert performance category (TP, FP, FN, or TN), classified using maximum temperature-based thresholds that defined heat advisories and warnings during 2012–2016, before the transition to the HeatRisk system. As in the main analyses, demographic subgroup differences were assessed for all-cause ED visits using the same interaction-based approach. Both analyses employed the same modeling structure, differing only in the exposure variable (alert issuance or alert performance). Overdispersion was assessed using the Pearson dispersion ratio, with values remaining below the 1.2 threshold recommended by Payne and colleagues [18], supporting the use of Poisson regression. Collinearity between alert status and maximum temperature within the fitted models was assessed using variance inflation factors (VIF), with scaled generalized VIF values below 1.10 for both alert status and the temperature spline in both the advisory and warning models, indicating no meaningful collinearity. Daily ED visit counts were modeled using Poisson regression with a log link:

$$\begin{aligned}&{Y}_{it}\sim\:{Poisson}\left({\mu\:}_{it}\right) \:{log}\left({\mu\:}_{it}\right)\\&={\alpha\:}_{i}+{\beta\:}_{1}{X}_{it}+{ns}({Tmax}_{it},3)\\&+{ns}({Doy}_{t},7)+\gamma\:{Dow}_{t}+\delta\:{Year}_{t} \end{aligned}$$

where $\:{Y}_{it}$ is the observed daily ED visit count for a ZIP code * i* on day * t*; $\:{\alpha\:}_{i}$ is a ZIP code fixed effect absorbing all time-invariant spatial confounding; ${ns}({Tmax}_{it},3)$ is a natural cubic spline of same-day maximum temperature with 3 degrees of freedom, capturing non-linear heat–health associations; ${ns}({Doy}_{t},7)$ is a natural cubic spline of day-of-year with 7 degrees of freedom, controlling for seasonality; ${Dow}_{t}$ is a categorical indicator for day of week, controlling for weekly variation in healthcare utilization; and ${Year}_{t}$ is a categorical indicator for calendar year, controlling for long-term temporal trends. The exposure term $\:{X}_{it}$ differs between analyses. In the main analyses, $\:{X}_{it}\in\:\left\{\mathrm{0,1}\right\}$ is a binary indicator of alert issuance (0 = No, 1 = Yes), modeled independently for advisory and warning; in the secondary analyses, $\:{X}_{it}\in\:\left\{{TN},\:{FP},\:{FN},\:{TP}\right\}$ corresponds to alert performance categories. In both cases, ${e}{x}\mathrm{p}\left({\beta\:}_{1}\right)$ is the relative risk (RR), reported as a percentage change in daily ED visit counts and calculated as ${RR}-1)\times\:100$, with corresponding 95% confidence intervals (CIs). In the secondary analyses, four contrasts were estimated: TP vs. TN, FP vs. TN, FN vs. TN, and TP vs. FN.

Since heat alerts are issued for specific days and are intended to prompt immediate behavioral responses, any association with ED visits was expected to be predominantly driven by same-day effects. Same-day (lag 0) maximum temperature was therefore controlled for, allowing effect estimates to be interpreted as the association between heat alerts and ED utilization beyond the direct physiological effects of heat. Although UC San Diego Health does not capture all ED visits in the county, the analytic approach relies on within-ZIP code temporal variation, which limits bias from incomplete population coverage provided catchment patterns remained stable over time.

Multiple sensitivity analyses were conducted to assess the robustness of the findings. First, all models were re-estimated after restricting the dataset to ZIP codes in which at least 80% of the population resided within the assigned climatic zone. Second, analyses were repeated after excluding ED visits from out-of-county residents. Third, to account for immediate and short-term delayed effects of heat, daily maximum temperature was modeled as two strata, with lag 0 specified using a natural cubic spline with 3 degrees of freedom and the mean of temperatures over lags 1–3 included as a linear term. Fourth, models were additionally adjusted for relative humidity. Fifth, to assess the potential influence of the transition from threshold-based alerts to the HeatRisk system, main analyses were repeated separately for the periods 2012–2016 and 2017–2023. Sixth, all models were re-estimated after restricting the dataset to ZIP codes within the Coastal climatic zone. Seventh, a distributed lag model (DLM) with alert issuance indicators at lags 0–3 was fitted to assess potential delayed effects on ED utilization.

All analyses were performed in R version 4.5.2 [19].

## Results

### Descriptive statistics

During the 2012–2023 period, a total of 443,106 all-cause ED visits were recorded among adults through the warm-season months (see Table 1). These visits originated from 113 unique ZIP codes, including 64,885 (14.6%) visits from individuals residing outside San Diego County. By climatic zone, 383,255 visits (86.5%) were from the Coastal region, 58,361 (13.2%) from Inland, 1,236 (0.28%) from the Mountains, and 254 (0.06%) from the Desert. This distribution reflects a pronounced decline in visits with increasing distance from UC San Diego Health emergency facilities.

**Table 1:** Summary statistics for ED visits by demographic characteristic and cause-specific categories

ED visits			
		*n* (%)	Median age, years (IQR)
Demographic characteristics			
Sex	Male	229,805 (51.9)	50 (35–62)
	Female	213,180 (48.1)	49 (33–63)
Ethnicity	Non-Hispanic	325,109 (75.5)	51 (35–64)
	Hispanic	105,646 (24.5)	44 (30–58)
Age groups	18–64 years	344,767 (77.8)	43 (30–54)
	65 + years	98,339 (22.2)	73 (68–81)
Cause-specific			
All-cause		443,106 (100)	50 (34–63)
Circulatory		24,686 (5.6)	62 (51–73)
Respiratory		19,712 (4.5)	53 (34–66)
Mental		31,713 (7.2)	43 (30–55)
Neurological		9,638 (2.2)	47 (33–60)
Digestive		30,234 (6.8)	51 (35–63)
Endocrine		10,591 (2.4)	55 (41–67)
Urinary		20,954 (4.7)	55 (38–69)
Accident		56,052 (12.7)	46 (30–62)
Heat-related		2,915 (0.66)	61 (46–73)

**Fig. 2:**
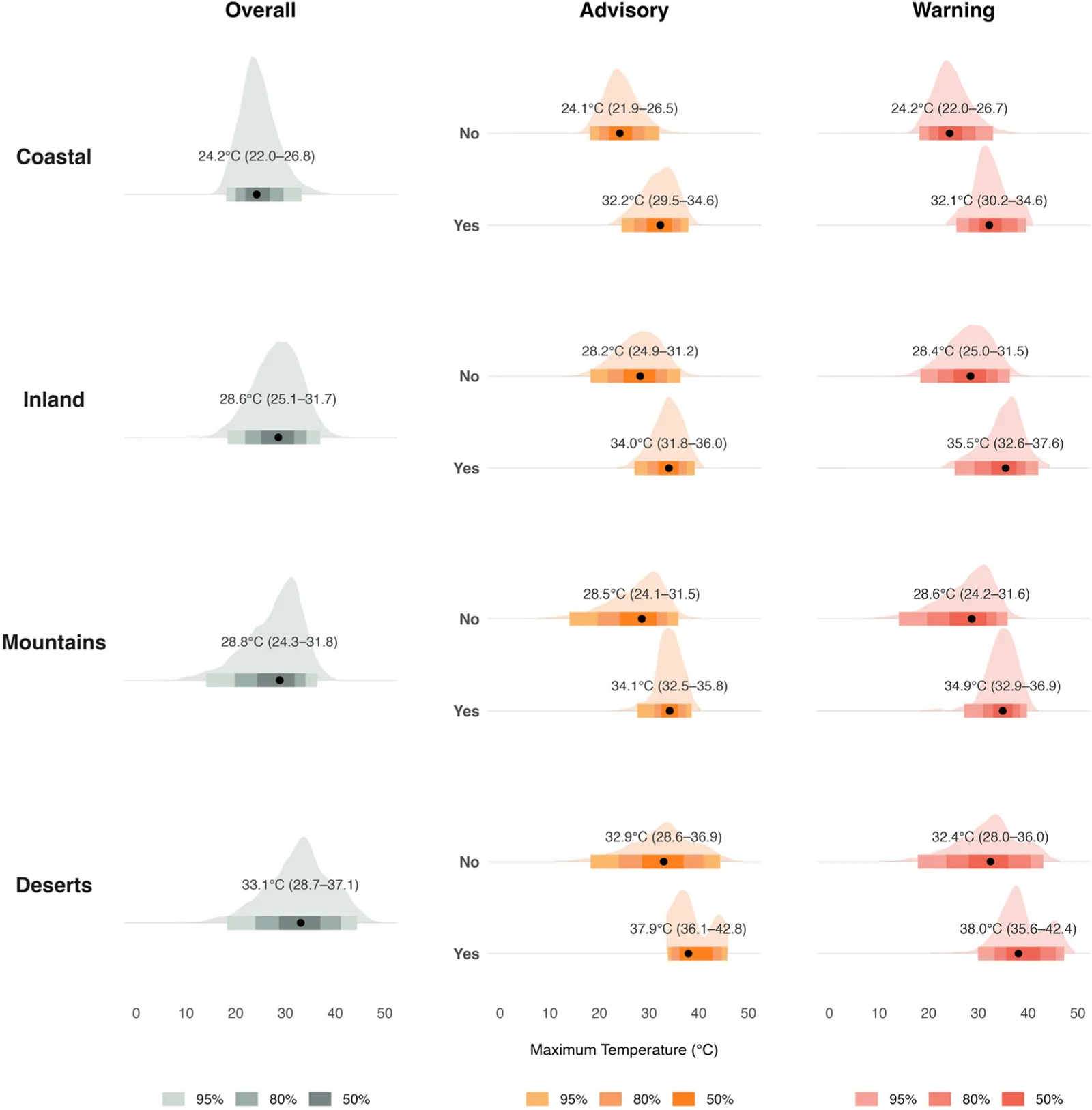
Distribution of maximum temperature by climatic zone and alert type, presenting median and IQR with 95%, 80%, and 50% central ranges, 2012–2023

As shown in Fig. 2, maximum temperature varied substantially across climatic zones and alert type categories. Coastal ZIP codes had the lowest maximum temperatures, with a median of 24.2 °C and a narrow IQR of 22.0–26.8 °C. Inland and Mountain zones showed higher values, each with median temperatures of approximately 28 °C. Desert areas recorded the highest temperatures, with a median of 33.1 °C and an IQR of 28.7–37.1 °C. Across all zones, alert days exhibited mostly left-skewed temperature distributions, with median maximum temperature values approximately 5–8 °C higher than on non-alert days. The monthly distribution of issued heat alerts by climatic zone is presented in Supplementary Figure S1 (advisories) and Figure S2 (warnings). During the 2012–2016 period, a total of 165,132 all-cause ED visits were recorded, of which 24,862 (15.1%) were from out-of-county residents.

Descriptive statistics for daily maximum temperature by heat alert performance category are presented in Table 2. Across both alert types, mean maximum temperature was higher on TP and FN days than on FP and TN days. Notably, among heat advisories, FN days exhibited the highest mean temperature (37.56 °C) of all four performance categories.

**Table 2:** Summary statistics for daily maximum temperature by heat alert performance categories, 2012–2016

Alert type	Performance	Mean	SD	5^th^ percentile	25^th^ percentile	50^th^ percentile	75^th^ percentile	95^th^ percentile
Advisory	TP	37.04	1.60	35.12	35.73	36.73	38.12	39.7
	FP	32.18	3.09	26.45	30.33	32.61	34.33	36.74
	FN	37.56	2.80	35.02	35.61	36.86	38.74	44.56
	TN	25.74	4.51	18.91	22.58	25.37	28.8	33.45
Warning	TP	40.40	2.49	37.91	38.6	39.67	41.46	47.16
	FP	34.20	4.12	27.04	31.55	34.43	36.86	40.14
	FN	38.57	0.99	37.78	38.03	38.4	39.01	40.6
	TN	26.00	4.68	18.97	22.69	25.56	29.17	34.09

**Fig. 3:**
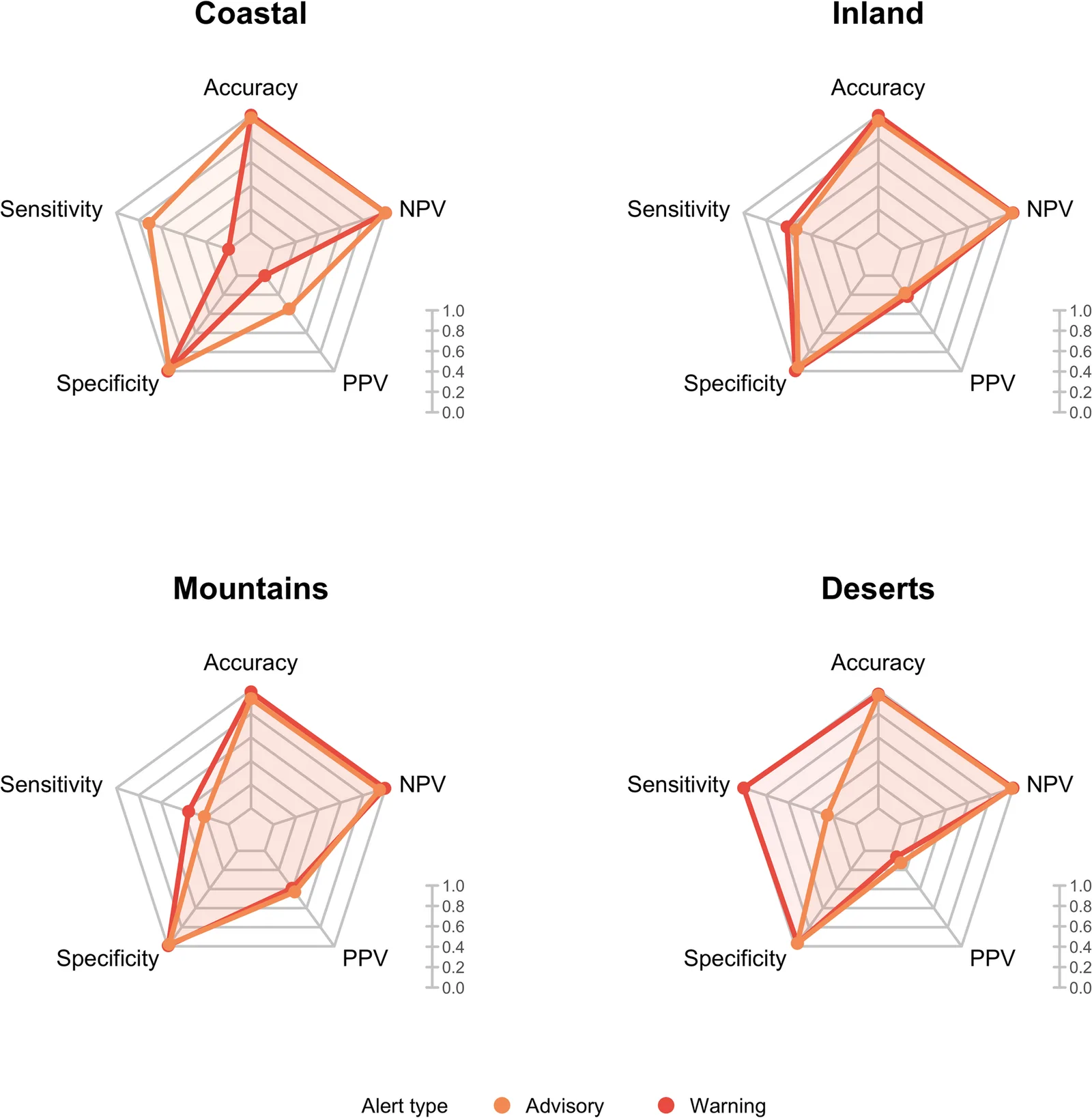
Radar plots of heat advisory and warning accuracy, sensitivity, specificity, PPV, and NPV across climatic zones, 2012–2016

The distribution of heat alert performance categories by climatic zone is presented in Table 3, providing the underlying counts and proportions that inform the diagnostic performance metrics reported in Fig. 3. In all zones, accuracy, specificity, and NPV were particularly high, reflecting the predominance of non-alert days, whereas sensitivity and PPV were consistently lower. Sensitivity for warnings ranged from zero in the Coastal zone, where no warnings were issued during this period, to modest values in the Inland (0.61) and Mountain (0.35) zones, and to perfect detection in the Desert zone (1.00). For the less severe advisories, sensitivity exceeded that of warnings only in the Coastal region (0.71), whereas it was consistently lower for Inland (0.53), Mountains (0.22), and Deserts (0.26). Overall, these metrics indicate that the system was highly effective at identifying non-alert days but demonstrated a more limited ability to detect true alert conditions across most zones.

**Table 3:** Distribution of heat alert performance categories by climatic zone, 2012–2016

Climatic zone	Alert	Performance	ZIP-Days	Percentage
Coastal	Warning	TP	0	0.00
		FP	0	0.00
		FN	117	0.19
		TN	60,131	99.81
	Advisory	TP	663	1.10
		FP	1241	2.06
		FN	271	0.45
		TN	58,073	96.39
Inland	Warning	TP	27	0.10
		FP	97	0.35
		FN	17	0.06
		TN	27,325	99.49
	Advisory	TP	261	0.95
		FP	1165	4.24
		FN	228	0.83
		TN	25,812	93.98
Mountains	Warning	TP	47	0.44
		FP	73	0.69
		FN	81	0.76
		TN	10,431	98.11
	Advisory	TP	160	1.50
		FP	212	1.99
		FN	554	5.21
		TN	9706	91.29
Deserts	Warning	TP	6	0.23
		FP	90	3.39
		FN	0	0.00
		TN	2562	96.39
	Advisory	TP	12	0.45
		FP	84	3.16
		FN	33	1.24
		TN	2529	95.15

### Heat alert issuance on ED visits

In the cause-specific analyses (see Table 4), heat advisory issuance was associated with a 2.1% increase in all-cause ED visits (95% CI: 0.4, 3.9), as well as increases in neurological (13.5; 95% CI: 1.3, 27.1) and heat-related visits (21.8; 95% CI: 1.6, 46.1). In contrast, heat warning issuance was associated only with an increase in heat-related visits (35.8; 95% CI: 3.5, 78.3). For all-cause ED visits, associations with both alert types were observed exclusively in females. Heat advisory issuance was associated with a 3.6% increase (95% CI: 1.2, 6.1) in daily ED visits among females, and heat warning issuance with a 4.7% increase (95% CI: 0.7, 8.7), with neither alert type showing an association in males. By ethnicity, advisory issuance was associated with a 2.9% increase in visits among non-Hispanic individuals (95% CI: 0.9, 4.9), with no corresponding association observed among Hispanics. With respect to age, heat advisory issuance was associated with marginal increases across both age groups, whereas heat warning issuance was associated with a pronounced increase restricted to adults aged 65 years and older (12.7; 95% CI: 6.8, 18.8).

**Table 4:** Percent change in ED visits associated with heat advisory and warning issuance, by cause and demographic characteristics

Alert type		Advisory		Warning	
		Percent change (95% CI)	*p*-value	Percent change (95% CI)	*p*-value
Cause-specific					
Circulatory		2.3 (-5.0, 10.0)	0.551	-2.8 (-13.6, 9.3)	0.635
Respiratory		1.9 (-6.4, 10.9)	0.667	12.8 (-1.5, 29.1)	0.082
Mental		0.1 (-6.4, 7.1)	0.979	-1.5 (-12.4, 10.9)	0.807
Neurological		13.5 (1.3, 27.1)*	0.029	-9.1 (-25.3, 10.6)	0.341
Digestive		-4.7 (-10.9, 1.9)	0.159	-0.3 (-10.3, 10.8)	0.959
Endocrine		8.2 (-2.9, 20.6)	0.154	-5.0 (-20.4, 13.5)	0.573
Urinary		6.4 (-1.5, 14.9)	0.116	2.5 (-9.5, 16.0)	0.700
Accident		4.3 (-0.8, 9.6)	0.099	5.8 (-2.5, 14.7)	0.174
Heat-related		21.8 (1.6, 46.1)*	0.033	35.8 (3.5, 78.3)*	0.027
All-cause		2.1 (0.4, 3.9) *	0.018	1.3 (-1.5, 4.2)	0.380
Sex	Male	0.7 (-1.6, 3.0)	0.552	-1.8 (-5.5, 2.0)	0.341
	Female	3.6 (1.2, 6.1)**	0.003	4.7 (0.7, 8.7)*	0.019
Ethnicity	Non-Hispanic	2.9 (0.9, 4.9)**	0.005	0.7 (-2.5, 4.0)	0.664
	Hispanic	-0.7 (-3.9, 2.6)	0.675	2.9 (-2.5, 8.5)	0.298
Age groups	18–64 years	1.8 (-0.2, 3.8)	0.071	-2.0 (-5.0, 1.2)	0.217
	65 + years	3.3 (-0.1, 6.9)	0.056	12.7 (6.8, 18.8)***	< 0.001

*p < 0.05; **p < 0.01; ***p < 0.001

### Heat alert performance on ED visits

**Fig. 4:**
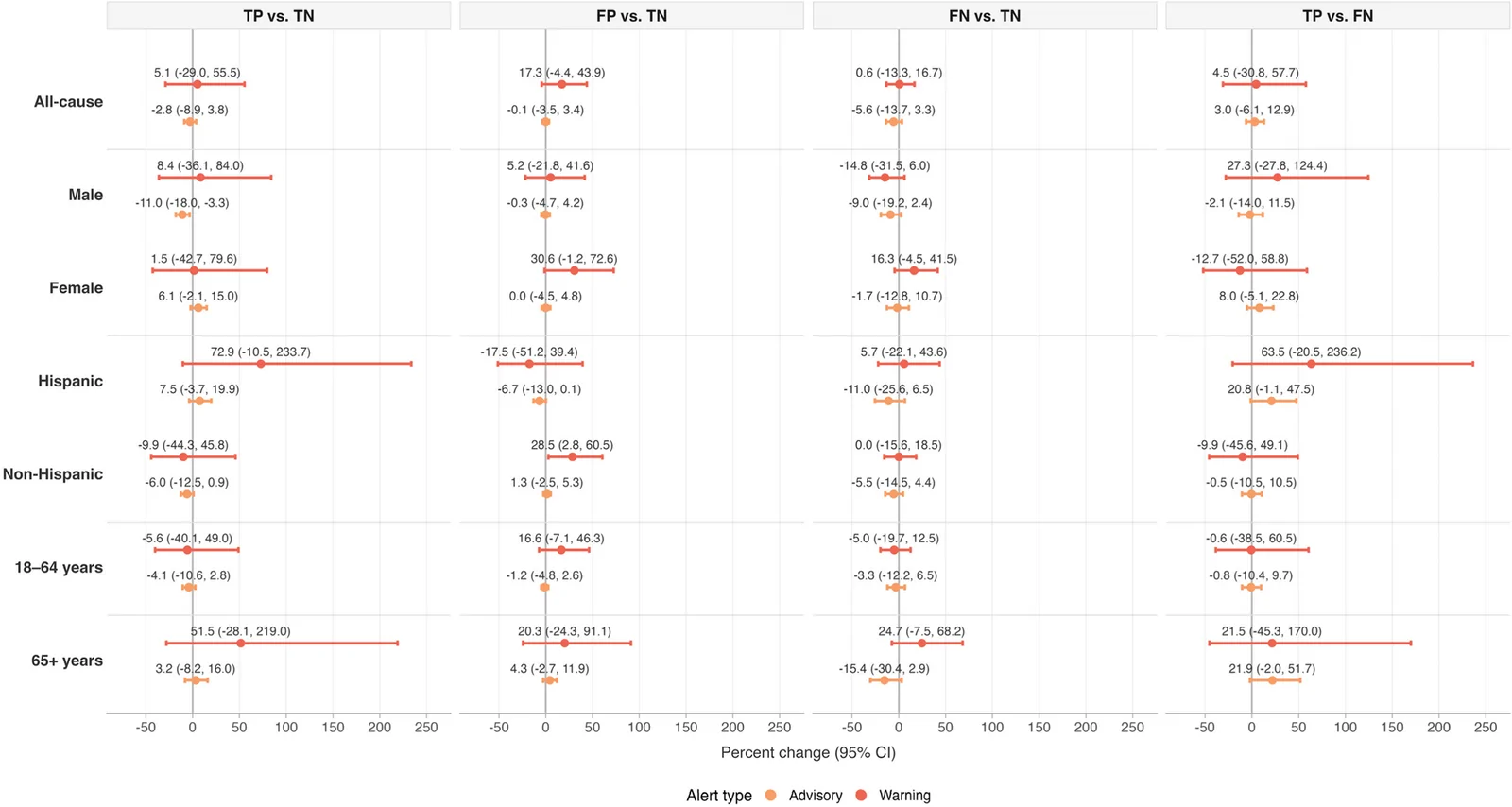
Percent change in ED visits associated with heat advisory and warning performance by demographic characteristics

Secondary analyses showed no consistent associations between alert performance categories and all-cause ED visits. The TP vs. TN contrast showed limited evidence of an overall effect, though heat advisory issuance on days when an alert was warranted was associated with an 11% reduction in ED visits among males (95% CI: -18.0, -3.3). Conversely, the FP vs. TN contrast for heat warnings was associated with a 28.5% increase in ED visits among non-Hispanic individuals (95% CI: 2.8, 60.5), with no corresponding association observed in other subgroups. Neither the FN vs. TN nor the TP vs. FN contrast was associated with differences in all-cause ED visits across demographic subgroups (Fig. 4).

### Sensitivity analyses

To evaluate the robustness of the findings, a series of sensitivity analyses were conducted using alternative sample restrictions and modeling specifications. Results were consistent with those of the main analysis, with no meaningful changes in the direction or magnitude of the estimated effects, when restricting the analysis to ZIP codes in which at least 80% of the population resided within the assigned climatic zone (Supplementary Table S3 and Figure S5), excluding ED visits from out-of-county residents (Supplementary Table S4 and Figure S6), incorporating a short-term temperature lag (Supplementary Table S5 and Figure S7), and additionally adjusting for relative humidity (Supplementary Table S6 and Figure S8). When analyses were stratified by period, associations were broadly consistent in direction with the main analysis. Heat-related ED visits showed the strongest associations in both periods, though the pattern differed by alert type. These period-specific findings should nevertheless be interpreted with caution given differences in statistical power and alert issuance criteria across periods (Supplementary Table S7). Restricting the analysis to ZIP codes within the Coastal climatic zone produced advisory associations that were broadly consistent in direction with those of the main analysis, although several estimates were attenuated and no longer statistically significant. Warning associated estimates showed greater instability, including a reversal in direction for females. Performance based contrasts for warnings could not be reliably estimated, as no warnings were issued during 2012 to 2016 in the Coastal zone (Supplementary Table S8 and Figure S9). In the distributed lag model sensitivity analysis, cumulative effects across lags 0–3 were statistically significant for advisories (4.0; 95% CI: 1.8, 6.3) and non-significant for warnings (1.9; 95% CI: -1.4, 5.3), consistent with the main findings in both direction and magnitude. No statistically significant lag-specific associations were observed for either alert type (Supplementary Table S9).

## Discussion

This study examined associations between NWS heat alert issuance, alert performance, and ED utilization in San Diego County, treating heat alerts as early warning signals intended to support timely public health action and risk notification within the operational context of the county’s EHRP. Overall, heat advisories and warnings were associated with limited changes in ED utilization, with effects primarily confined to heat-related conditions and to the most susceptible population groups, corresponding to an estimated 346 excess advisory-related and 76 excess warning-related all-cause ED visits over the study period. Distinct patterns emerged by alert type. Heat warnings, which predominantly coincide with periods of extreme heat, were associated exclusively with increased heat-related ED visits and with higher all-cause ED utilization among females and older adults, suggesting that residual and clinically relevant health effects persist among the most heat-sensitive conditions and populations during periods of extreme heat. Heat advisories, issued more frequently and particularly prevalent in the Coastal zone, where most visits originated, were associated with broader increases in ED utilization, including neurological, heat-related, and all-cause ED visits. Among demographic subgroups, higher all-cause ED utilization following advisory issuance was observed among females and non-Hispanic individuals, with marginal increases across both age groups.

In the Coastal zone, greater frequency of advisories may have attenuated perceived risk over time, potentially reducing the consistency of protective behaviors among the population. The heightened ED utilization observed among older adults following heat warning issuance is consistent with their well-documented susceptibility to heat-related morbidity, driven by age-related declines in thermoregulation that reduce the ability to dissipate excess heat. This susceptibility is further compounded by a higher prevalence of chronic conditions, use of medications that interfere with thermoregulatory function, and functional limitations that may restrict adaptive responses to heat [1]. Additionally, older adults are more likely to reside in housing with inadequate ventilation or without air conditioning [20], collectively limiting their ability to adopt protective behaviors in response to heat alerts. Under the current EHRP, advisories and warnings correspond to distinct activation tiers, Phase II and Phase III respectively [9], reflecting a formally higher institutional response threshold for warnings. However, the intensity of resource mobilization by alert type, such as the number of activated cooling centers or outreach efforts undertaken, is not specified in available EHRP documentation, precluding direct evaluation of whether differential community response contributed to the observed differences between advisories and warnings.

Heat advisory issuance on days when conditions warranted an alert (TP vs. TN) was associated with a reduction in all-cause ED visits among males, whereas false positive warning issuance (FP vs. TN) was associated with an increase in all-cause ED visits among non-Hispanic individuals. No associations were observed for missed alerts (FN vs. TN) or for correctly issued alerts relative to missed alerts (TP vs. FN), across any demographic subgroup. This suggests that the role of individual personal heat perception and adaptive behaviors cannot be excluded from the observed associations. Consequently, differences between performance categories may reflect both the alert’s informational effect and unmeasured behavioral responses to heat that require further investigation through behavioral research. At the subgroup level, the modest reduction in ED visits among males following heat advisory issuance may reflect heightened awareness of heat-related risks in occupationally exposed settings, where outdoor and physically demanding work is more prevalent among men [21]. The increase observed among non-Hispanic individuals following false positive warning issuance may be more plausibly attributable to residual heat exposure than to warning issuance itself. False positive warning days were characterized by substantially higher maximum temperatures than true negative days despite not meeting alert thresholds. Further, the distributed lag analysis showed a significant cumulative effect for advisories in the absence of any single significant lag, suggesting a sustained influence on ED utilization over the days following issuance. The corresponding null cumulative effect for warnings may reflect their lower frequency and consequently reduced statistical power, rather than a true absence of delayed effects.

Although morbidity outcomes are considered more sensitive indicators of the health impacts of extreme heat, evaluations of heat-health action plans (HHAPs) and heat-health warning systems (HHWSs) have largely focused on mortality. Following the 2003 European heatwave [22], several countries established national HHAPs [23]. Time series analyses with distributed lag non-linear models comparing periods before and after their implementation reported reductions in heat-related mortality in Italy and Spain [24, 25], while a predictive modelling study in France estimated that the establishment of a national heat prevention plan may have resulted in approximately 4,400 deaths averted during the 2006 heatwave [26]. In the United States, a multiple linear regression analysis estimated that approximately 117 excess deaths were prevented in Philadelphia between 1995 and 1998 following the introduction of a HHWS [27]. In Australia, a case-series study from Adelaide found no evidence of reduced mortality following the implementation of a HHWS but, consistent with our findings, reported a significant reduction in heat-related morbidity [28].

To address the limitations of observational designs susceptible to confounding from secular trends, more recent studies have adopted quasi-experimental approaches. In Canada, a difference-in-differences analysis found that the introduction of a HHAP in Montreal in 2004 was associated with reduced mortality on hot days, with larger benefits observed among older adults and socioeconomically disadvantaged populations [29]. Building on this approach, Benmarhnia and colleagues [30] applied a similar difference-in-differences design in New York City, and found that lowering the heat alert threshold in 2008 was associated with a reduction in hospital admissions for heat-related illnesses among Medicare beneficiaries aged 65 years and older.

Studies examining the health effects of alert issuance specifically are more limited but particularly relevant to the present analysis. Two studies by Weinberger and colleagues evaluated the health effects of NWS heat alerts by comparing alert and non-alert days with similar temperatures using a matching approach. The first study found no overall reduction in all-cause mortality associated with heat alerts across 20 United States cities, with the exception of Philadelphia [13]. The second study, focused on Medicare beneficiaries aged 65 years and older, similarly found no mortality reductions associated with NWS heat alerts but observed higher rates of hospitalization for fluid and electrolyte disorders and heat stroke [12]. This pattern of heat-specific morbidity effects among older adults is broadly consistent with the associations observed in the present study.

The absence of mortality effects may partly reflect the heterogeneity of NWS-based alert systems across local offices in the United States, which vary considerably in their threshold criteria and degree of integration within local response plans [13]. As previously noted, In San Diego, NWS heat alerts trigger EHRP activation, guiding coordinated institutional responses including the opening of cooling resources and public health communications [9]. Comparisons with international evidence require additional caution, as HHWSs in other settings, particularly in Europe, are often embedded within broader HHAPs incorporating epidemiologically derived thresholds, surveillance and monitoring components, and multi-sectoral coordination, although the degree of integration and implementation varies substantially across countries [23, 31, 32]. Consequently, health benefits observed in those countries likely reflect the combined effect of HHWS issuance and accompanying HHAP measures rather than the alert signal alone, limiting direct comparability with the present findings.

The findings of the present study are broadly consistent with existing evidence indicating that associations between HHWSs and health outcomes are more frequently observed for morbidity than for mortality, particularly among older adults and for heat-specific conditions, reinforcing the importance of evaluating HHWSs beyond mortality. Whereas prior studies employing pre–post, quasi-experimental, or matched designs have primarily aimed to estimate the causal effectiveness of HHAPs and HHWSs as public health interventions, the present study addresses a complementary research question, examining variation in ED utilization associated with NWS heat alert issuance and performance within the operational context of San Diego County’s EHRP. As with other observational studies, the present design precludes causal interpretation. Nevertheless, these findings are relevant to both practitioners and policy makers, as they underscore how NWS heat alerts function as public communication signals influencing health risk and healthcare-seeking behavior, and how alert criteria and implementation shape observed morbidity patterns.

The present study has several methodological strengths. First, the study provides a comprehensive examination of the associations between heat alert issuance, alert performance, and ED utilization over a twelve-year period, and is, to our knowledge, one of the few to jointly assess both dimensions. The availability of detailed ED data enabled the examination of cause-specific visits as well as stratified analyses of all-cause visits by demographic characteristics, including sex, ethnicity, and age, providing insight into differential vulnerabilities and enhancing relevance for targeted public health planning. Furthermore, San Diego County represents a unique setting with four distinct climate zones, allowing examination of variation in NWS heat alert system performance across different climatic contexts. Alert performance was highest in the desert zone, where heat warnings demonstrated maximal sensitivity, suggesting that uniform alert strategies may not perform equally across diverse climates and indicating potential benefits of climate-specific approaches.

The present study is subject to several limitations. First, contextual factors that may modify vulnerability to heat-related morbidity, including access to air-conditioning, availability and utilization of cooling centers, housing characteristics, and occupational heat exposure, could not be accounted for due to data limitations. Community and public health responses to heat events likely evolved over the study period, though such changes are not expected to have introduced systematic bias across the study population. Second, the COVID-19 pandemic occurred during part of the study period and may have influenced ED utilization independently of heat alert issuance. Although unlikely to have systematically biased alert-specific associations, its broader influence on utilization patterns cannot be excluded. Third, analyses were conducted at the ZIP code level using aggregated daily counts rather than individual-level data, and findings may therefore be subject to ecological bias. Fourth, behavioral mechanisms through which heat alerts may influence health outcomes were not directly measured, and observed associations may reflect a combination of physiological responses to heat and behavioral responses to alert issuance. Fifth, performance analyses were restricted to the 2012–2016 period due to limited availability of HeatRisk variables, which may have reduced statistical power. Because alert performance classification depends on the threshold criteria in effect during this period, and these differ from those used under the current HeatRisk system, the generalizability of these findings to present-day alert operations may be limited. Sixth, because heat alerts are issued largely in response to forecasted conditions, alert status is inherently correlated with temperature (Supplementary Figure S3). Although the models adjust for maximum temperature, alert status may retain forecast information not captured by this adjustment. The reported associations may therefore reflect both a direct effect of alert issuance and residual confounding by forecast information, which the present design cannot disentangle. Finally, ED data were obtained exclusively from UC San Diego Health facilities, located in the Coastal zone, which may limit generalizability and precluded stratification of health outcome analyses by climatic zone. Consequently, the extent to which alert effectiveness varies across environmental contexts remains unaddressed. The restriction of analyses to adults introduces a further constraint on external validity, as children are also recognized as a heat-vulnerable population.

## Conclusion

This study examined associations between heat alert issuance, alert performance, and ED utilization in San Diego County. Overall, heat alerts were associated with limited changes in ED visit counts, with effects largely confined to heat-related conditions and to susceptible population groups. Distinct patterns emerged by alert type. Heat advisories were associated with broader increases in ED utilization, including neurological, heat-related, and all-cause visits, with higher all-cause utilization observed among females and non-Hispanic individuals. In contrast, heat warnings were associated with increased heat-related ED visits and higher all-cause ED utilization among females and older adults, suggesting that clinically relevant health effects persist among the most heat-sensitive populations even when alerts are issued. Alert performance analyses provided limited overall evidence of differential ED utilization. Taken together, these findings indicate that heat alerts function as public health communication signals with varying effects across alert type and demographic group, and that the behavioral mechanisms through which alerts influence health outcomes remain incompletely characterized. Continued evaluation of HHWSs across climatic zones and demographic subgroups, incorporating behavioral and contextual data, is necessary to better understand how alert design and implementation can be optimized to reduce heat-related morbidity.

## Supplementary Information

Supplementary Material 1.

## Data Availability

The data used in this study is confidential.
