# Supplementary material for: Heat alerts and emergency department utilization: evidence from San Diego County, California
Source: Environ Health. 2026 Jul 28;25:82. doi: 10.1186/s12940-026-01325-4 (PMC13711694; doi:10.1186/s12940-026-01325-4)

Heat alerts and emergency department utilization: evidence from San Diego County, California

Leire Luque-García[a, *], Oghenetanure R. Enaworu[b], William Bruno[c], Edward Castillo[c], Theodore C. Chan[c], Wael K. Al-Delaimy[a]

a. Herbert Wertheim School of Public Health and Human Longevity Science, University of California San Diego, La Jolla, CA, United States.

b. Chao Family Comprehensive Cancer Center, University of California Irvine, Irvine, CA, United States.

c. Department of Emergency Medicine, University of California San Diego, La Jolla, CA, United States.

* Corresponding author: lluquegarcia@health.ucsd.edu

Supplementary Material

Text S1: Description of climatic zones in San Diego County. 2
Text S2: Definitions and formulas of diagnostic performance metrics for heat alerts. 3
Table S1: International Classification of Diseases (ICD) codes used to classify cause-specific ED visits. 4
Table S2: Maximum temperature thresholds by climatic zone and month. 5
Table S3: Sensitivity analysis restricting to ZIP codes in which at least 80% of the population resided within the assigned climatic zone. 6
Table S4: Sensitivity analysis excluding out-of-county residents. 7
Table S5: Sensitivity analysis incorporating a short-term temperature lag. 8
Table S6: Sensitivity analysis controlling for relative humidity 9
Table S7: Sensitivity analysis stratified by period 2012–2016 and 2017–2023. 10
Table S8: Sensitivity analysis restricting to the Coastal climatic zone. 11
Table S9: Sensitivity analysis using a distributed lag model, by lag day and cumulative effect across lags 0–3. 12
Figure S1: Monthly number of heat advisories issued by climatic zone. 13
Figure S2: Monthly number of heat warnings issued by climatic zone. 14
Figure S3: Pearson correlation coefficients between heat alert issuance. 15
Figure S4: Geographic assignment of ZIP codes to climatic zones based on population distribution. 16
Figure S5: Sensitivity analysis restricting to ZIP codes in which at least 80% of the population resided within the assigned climatic zone. 17
Figure S6: Sensitivity analysis excluding out-of-county residents. 18
Figure S7: Sensitivity analysis incorporating a short-term temperature lag. 19
Figure S8: Sensitivity analysis controlling for relative humidity. 20
Figure S9: Sensitivity analysis restricting to the Coastal climatic zone. 21

Text S1: Description of climatic zones in San Diego County.

Coastal: This zone is characterized by mild, stable temperatures and a strong oceanic influence. A persistent marine layer produces consistently cool, humid conditions that moderate temperature extremes throughout the year. Summers are warm but rarely hot, while winters remain generally mild.

Inland: This zone is characterized by hot, dry summers and mild winters with limited precipitation. The reduced influence of the ocean contributes to significant diurnal and seasonal temperature variability, with high daytime temperatures and lower relative humidity. Atmospheric conditions, with minimal cloud cover and low moisture content, produce consistently high levels of solar radiation throughout the year.

Mountains: is zone encompasses higher-elevation areas with distinct climatic characteristics, including cooler temperatures and greater precipitation relative to lower-lying zones. This zone has a pronounced seasonal variability, with warm summers and cold winters that frequently produce cold temperatures and occasional snowfall. The combined effects of higher altitude, inland location, and reduced atmospheric moisture result in substantial diurnal temperature fluctuations.

Deserts: This zone experiences the most extreme temperature conditions in the county, with intensely hot, arid summers and relatively mild winters. Summer daytime temperatures frequently exceed 37.8 ºC (100 °F), while nights experience substantial cooling that creates pronounced daily temperature variations. This climate is further defined by persistently low humidity levels and minimal annual precipitation.

Text S2: Definitions and formulas of diagnostic performance metrics for heat alerts.

The diagnostic performance of heat alerts was assessed using five standard metrics, namely accuracy, sensitivity, specificity, positive predictive value (PPV), and negative predictive value (NPV), defined as follows:

- **Accuracy:** the proportion of correctly classified days.
    - Formula: (TP+TN)/(TP+TN+FP+FN)
- **Sensitivity:** the proportion of threshold-exceedance days on which an alert was correctly issued.
    - Formula: TP/(TP+FN)
- **Specificity:** the proportion of non-threshold-exceedance days correctly classified.
    - Formula: TN/(TN+FP)
- **PPV:** the proportion of alert days that corresponded to threshold exceedances.
    - Formula: TP/(TP+FP)
- **NPV:** the proportion of non-alert days that corresponded to non-threshold exceedances.
    - Formula: TN/(TN+FN)

Table S1: International Classification of Diseases (ICD) codes used to classify cause-specific ED visits.

Cause specific	ICD-9	ICD-10
Circulatory	390–459	I00–I99, G45
Respiratory	460–519	J00–J99
Mental health	290–319	F00–F99
Neurological	320–359	G00–G99
Digestive	520–579	K00–K95
Endocrine	240–279	E00–E89
Urinary	580–599	N00–N39
Accident	800–959	S00–S99, T00–T35, V00–V99, W00–W99, X00–X59
Heat-related	992, E900	T67, X30, E86, E87.1

Table S2: Maximum temperature thresholds by climatic zone and month.

Climatic zone	Alert	January	February	March	April	May	June	July	August	September	October	November	December
**Coastal**	Warning	100°F (37.8°C)	100°F (37.8°C)	100°F (37.8°C)	100°F (37.8°C)	100°F (37.8°C)	100°F (37.8°C)	100°F (37.8°C)	100°F (37.8°C)	100°F (37.8°C)	100°F (37.8°C)	100°F (37.8°C)	100°F (37.8°C)
	Advisory	95°F (35.0°C)	95°F (35.0°C)	95°F (35.0°C)	95°F (35.0°C)	95°F (35.0°C)	95°F (35.0°C)	95°F (35.0°C)	95°F (35.0°C)	95°F (35.0°C)	95°F (35.0°C)	95°F (35.0°C)	95°F (35.0°C)
**Inland**	Warning	105°F (40.6°C)	105°F (40.6°C)	105°F (40.6°C)	105°F (40.6°C)	105°F (40.6°C)	105°F (40.6°C)	105°F (40.6°C)	105°F (40.6°C)	105°F (40.6°C)	105°F (40.6°C)	105°F (40.6°C)	105°F (40.6°C)
	Advisory	100°F (37.8°C)	100°F (37.8°C)	100°F (37.8°C)	100°F (37.8°C)	100°F (37.8°C)	100°F (37.8°C)	100°F (37.8°C)	100°F (37.8°C)	100°F (37.8°C)	100°F (37.8°C)	100°F (37.8°C)	100°F (37.8°C)
**Mountains**	Warning	95°F (35.0°C)	95°F (35.0°C)	95°F (35.0°C)	95°F (35.0°C)	95°F (35.0°C)	100°F (37.8°C)	100°F (37.8°C)	100°F (37.8°C)	100°F (37.8°C)	95°F (35.0°C)	95°F (35.0°C)	95°F (35.0°C)
	Advisory	90°F (32.2°C)	90°F (32.2°C)	90°F (32.2°C)	90°F (32.2°C)	90°F (32.2°C)	95°F (35.0°C)	95°F (35.0°C)	95°F (35.0°C)	95°F (35.0°C)	90°F (32.2°C)	90°F (32.2°C)	90°F (32.2°C)
**Deserts**	Warning	113°F (45.0°C)	113°F (45.0°C)	113°F (45.0°C)	113°F (45.0°C)	113°F (45.0°C)	115°F (46.1°C)	117°F (47.2°C)	117°F (47.2°C)	115°F (46.1°C)	115°F (46.1°C)	113°F (45.0°C)	113°F (45.0°C)
	Advisory	108°F (42.2°C)	108°F (42.2°C)	108°F (42.2°C)	108°F (42.2°C)	108°F (42.2°C)	112°F (44.4°C)	112°F (44.4°C)	112°F (44.4°C)	112°F (44.4°C)	110°F (43.3°C)	108°F (42.2°C)	108°F (42.2°C)

Table S3: Sensitivity analysis restricting to ZIP codes in which at least 80% of the population resided within the assigned climatic zone. Percent change in ED visits associated with heat advisory and warning issuance, by cause and demographic characteristics. Note: * $p < 0.05$; ** $p < 0.01$; *** $p < 0.001$.

Alert type		Advisory		Warning	
		Percent change (95% CI)	p-value	Percent change (95% CI)	p-value
**Cause-specific**					
Circulatory		5.1 (-2.5, 13.4)	0.196	-6.9 (-17.9, 5.5)	0.263
Respiratory		2.9 (-5.7, 12.2)	0.520	14.9 (0.0, 32.0)	0.051
Mental		0.1 (-6.5, 7.3)	0.966	-2.5 (-13.7, 10.1)	0.678
Neurological		16.0 (3.2, 30.5)*	0.013	-10.3 (-27.0, 10.3)	0.303
Digestive		-4.6 (-11.1, 2.3)	0.183	0.8 (-9.7, 12.5)	0.892
Endocrine		6.3 (-5.0, 18.9)	0.288	-5.0 (-21.0, 14.2)	0.583
Urinary		7.7 (-0.6, 16.7)	0.071	0.2 (-12.2, 14.3)	0.977
Accident		5.2 (-0.1, 10.7)	0.054	5.1 (-3.4, 14.3)	0.251
Heat-related		18.0 (-2.5, 42.9)	0.089	32.2 (-0.9, 76.3)	0.058
**All-cause**		2.5 (0.7, 4.4)**	0.007	0.5 (-2.4, 3.5)	0.724
Sex	Male	1.1 (-1.3, 3.5)	0.362	-2.3 (-6.1, 1.7)	0.254
	Female	4.0 (1.5, 6.5)**	0.002	3.7 (-0.4, 7.9)	0.078
Ethnicity	Non-Hispanic	3.3 (1.2, 5.4)**	0.002	0.0 (-3.3, 3.4)	0.983
	Hispanic	-0.5 (-3.8, 3.0)	0.782	2.5 (-3.1, 8.3)	0.392
Age groups	18-64 years	2.1 (0.1, 4.2)*	0.040	-2.9 (-6.1, 0.3)	0.077
	65+ years	3.8 (0.3, 7.5)*	0.034	12.8 (6.7, 19.2)***	<0.001

Table S4: Sensitivity analysis excluding out-of-county residents. Percent change in ED visits associated with heat advisory and warning issuance, by cause and demographic characteristics. Note: * p < 0.05; ** p < 0.01; *** p < 0.001.

**Alert type**		**Advisory**		**Warning**	
		**Percent change (95% CI)**	**p-value**	**Percent change (95% CI)**	**p-value**
**Cause-specific**					
Circulatory		1.5 (-6.3, 9.9)	0.716	-0.7 (-12.3, 12.4)	0.907
Respiratory		2.1 (-6.8, 11.8)	0.654	7.8 (-6.8, 24.7)	0.311
Mental		0.1 (-6.9, 7.6)	0.980	-3.4 (-14.8, 9.6)	0.593
Neurological		14.9 (1.8, 29.8)*	0.025	-9.9 (-26.8, 10.8)	0.324
Digestive		-3.7 (-10.4, 3.5)	0.309	3.1 (-7.7, 15.3)	0.586
Endocrine		9.2 (-2.8, 22.7)	0.139	-6.0 (-22.2, 13.6)	0.521
Urinary		5.3 (-3.1, 14.5)	0.225	5.1 (-7.7, 19.6)	0.455
Accident		6.8 (1.2, 12.8)*	0.017	4.4 (-4.4, 13.9)	0.338
Heat-related		26.5 (4.2, 53.5)*	0.017	23.2 (-8.5, 66.0)	0.170
**All-cause**		2.7 (0.8, 4.6)**	0.005	1.1 (-1.8, 4.2)	0.460
Sex	Male	0.8 (-1.7, 3.3)	0.524	-2.6 (-6.4, 1.4)	0.200
	Female	4.7 (2.1, 7.4)***	<0.001	5.2 (1.1, 9.5)*	0.014
Ethnicity	Non-Hispanic	4.0 (1.8, 6.2)***	<0.001	1.3 (-2.1, 4.9)	0.446
	Hispanic	-1.5 (-4.9, 2.1)	0.407	0.9 (-4.6, 6.8)	0.755
Age groups	18-64 years	2.2 (0.1, 4.3)*	0.040	-2.6 (-5.9, 0.7)	0.120
	65+ years	4.4 (0.8, 8.3)*	0.017	14.4 (8.2, 20.9)***	<0.001

Table S5: Sensitivity analysis incorporating a short-term temperature lag. Percent change in ED visits associated with heat advisory and warning issuance, by cause and demographic characteristics. Note: * p < 0.05; ** p < 0.01; *** p < 0.001.

**Alert type**		**Advisory**		**Warning**	
		**Percent change (95% CI)**	**p-value**	**Percent change (95% CI)**	**p-value**
**Cause-specific**					
Circulatory		3.3 (-4.0, 11.3)	0.383	-1.6 (-12.6, 10.7)	0.787
Respiratory		2.0 (-6.4, 11.1)	0.657	12.9 (-1.4, 29.4)	0.080
Mental		-1.5 (-8.0, 5.4)	0.661	-3.1 (-13.9, 9.1)	0.602
Neurological		12.4 (0.2, 26.0)*	0.046	-10.4 (-26.4, 9.0)	0.273
Digestive		-4.6 (-10.9, 2.1)	0.171	-0.1 (-10.2, 11.1)	0.989
Endocrine		6.8 (-4.3, 19.1)	0.242	-6.7 (-21.9, 11.6)	0.448
Urinary		5.6 (-2.4, 14.1)	0.174	1.5 (-10.4, 15.0)	0.819
Accident		4.0 (-1.1, 9.4)	0.130	5.4 (-2.9, 14.3)	0.206
Heat-related		16.9 (-2.7, 40.4)	0.096	28.9 (-2.0, 69.4)	0.069
**All-cause**		1.9 (0.1, 3.7)*	0.036	1.0 (-1.8, 3.9)	0.503
Sex	Male	0.6 (-1.7, 3.0)	0.611	-2.0 (-5.6, 1.8)	0.309
	Female	3.5 (1.0, 6.0)**	0.005	4.5 (0.5, 8.6)*	0.025
Ethnicity	Non-Hispanic	2.9 (0.9, 5.0)**	0.005	0.7 (-2.5, 4.0)	0.682
	Hispanic	-0.7 (-3.9, 2.6)	0.675	2.8 (-2.5, 8.5)	0.304
Age groups	18-64 years	1.6 (-0.4, 3.6)	0.118	-2.2 (-5.3, 0.9)	0.166
	65+ years	3.0 (-0.4, 6.6)	0.084	12.3 (6.5, 18.4)***	<0.001

Table S6: Sensitivity analysis controlling for relative humidity. Percent change in ED visits associated with heat advisory and warning issuance, by cause and demographic characteristics. Note: * p < 0.05; ** p < 0.01; *** p < 0.001.

Alert type		Advisory		Warning	
		**Percent change (95% CI)**	**p-value**	**Percent change (95% CI)**	**p-value**
**Cause-specific**					
Circulatory		2.5 (-4.8, 10.3)	0.511	-3.1 (-13.8, 9.0)	0.603
Respiratory		1.6 (-6.7, 10.6)	0.717	13.2 (-1.2, 29.6)	0.073
Mental		0.3 (-6.3, 7.3)	0.938	-1.7 (-12.6, 10.7)	0.780
Neurological		13.4 (1.2, 27.1)*	0.030	-9.0 (-25.2, 10.7)	0.346
Digestive		-4.4 (-10.6, 2.3)	0.192	-0.7 (-10.7, 10.4)	0.892
Endocrine		8.3 (-2.8, 20.7)	0.149	-5.1 (-20.6, 13.4)	0.564
Urinary		6.6 (-1.3, 15.2)	0.105	2.2 (-9.7, 15.8)	0.726
Accident		4.4 (-0.7, 9.8)	0.091	5.7 (-2.6, 14.6)	0.182
Heat-related		22.4 (2.1, 46.8)*	0.029	35.3 (3.0, 77.6)*	0.030
**All-cause**		2.2 (0.4, 4.0)*	0.015	1.2 (-1.6, 4.1)	0.402
Sex	Male	0.8 (-1.5, 3.1)	0.523	-1.9 (-5.5, 1.9)	0.327
	Female	3.7 (1.3, 6.2)**	0.003	4.6 (0.7, 8.7)*	0.021
Ethnicity	Non-Hispanic	3.0 (0.9, 5.0)**	0.004	0.6 (-2.6, 3.9)	0.697
	Hispanic	-0.6 (-3.9, 2.7)	0.704	2.8 (-2.5, 8.5)	0.310
Age groups	18-64 years	1.8 (-0.1, 3.8)	0.064	-2.0 (-5.1, 1.1)	0.205
	65+ years	3.4 (0.0, 6.9)	0.052	12.6 (6.8, 18.7)***	<0.001

Table S7: Sensitivity analysis stratified by period 2012–2016 and 2017–2023. Percent change in ED visits associated with heat advisory and warning issuance, by cause and demographic characteristics. Note: * p < 0.05; ** p < 0.01; *** p < 0.001.

Alert type		Advisory		Warning	
		Percent change (95% CI)	p-value	Percent change (95% CI)	p-value
**2012-2016**					
**Cause-specific**					
Circulatory		12.4 (-1.9, 28.9)	0.093	43.1 (-29.9, 192.0)	0.325
Respiratory		-0.3 (-14.2, 15.8)	0.968	-42.4 (-81.7, 81.1)	0.345
Mental		1.0 (-10.2, 13.6)	0.865	10.3 (-51.1, 149.1)	0.813
Neurological		6.2 (-13.2, 29.9)	0.559	31.8 (-52.0, 261.4)	0.592
Digestive		-7.8 (-18.8, 4.8)	0.213	8.4 (-46.6, 119.9)	0.823
Endocrine		13.4 (-7.1, 38.3)	0.216	-12.9 (-72.7, 177.3)	0.815
Urinary		10.0 (-4.1, 26.2)	0.173	-16.5 (-65.8, 103.7)	0.692
Accident		-3.9 (-12.3, 5.4)	0.398	3.9 (-41.6, 84.9)	0.896
Heat-related		70.9 (25.3, 133.1)***	<0.001	-100.0 (-100.0, Inf)	0.990
**All-cause**		0.0 (-3.1, 3.3)	0.976	14.5 (-4.7, 37.5)	0.147
Sex	Male	-2.4 (-6.3, 1.7)	0.244	6.1 (-18.2, 37.6)	0.658
	Female	2.7 (-1.5, 7.0)	0.216	23.8 (-3.8, 59.2)	0.097
Ethnicity	Non-Hispanic	0.3 (-3.2, 3.9)	0.858	19.4 (-2.6, 46.4)	0.087
	Hispanic	-1.7 (-7.4, 4.4)	0.584	3.7 (-31.2, 56.4)	0.862
Age groups	18-64 years	-1.1 (-4.5, 2.4)	0.535	11.5 (-9.2, 36.8)	0.299
	65+ years	5.0 (-1.2, 11.6)	0.117	27.7 (-13.9, 89.3)	0.225
**2017-2023**					
**Cause-specific**					
Circulatory		-3.8 (-12.0, 5.2)	0.398	-2.5 (-14.0, 10.5)	0.691
Respiratory		1.4 (-8.6, 12.5)	0.798	9.5 (-5.4, 26.7)	0.223
Mental		-0.2 (-8.2, 8.6)	0.971	-2.8 (-14.4, 10.4)	0.665
Neurological		12.8 (-2.0, 29.7)	0.093	-6.3 (-24.1, 15.6)	0.542
Digestive		-4.8 (-12.2, 3.1)	0.225	-6.1 (-16.1, 5.2)	0.278
Endocrine		2.3 (-10.4, 16.7)	0.741	-5.7 (-21.9, 13.9)	0.541
Urinary		3.2 (-6.2, 13.6)	0.513	3.6 (-9.4, 18.3)	0.608
Accident		7.1 (0.8, 13.8)*	0.025	3.4 (-5.2, 12.8)	0.452
Heat-related		-1.4 (-22.1, 24.8)	0.910	49.3 (11.7, 99.5)**	0.007
**All-cause**		1.6 (-0.5, 3.8)	0.131	-0.2 (-3.1, 2.9)	0.901
Sex	Male	0.8 (-2.0, 3.7)	0.562	-2.8 (-6.6, 1.1)	0.156
	Female	2.5 (-0.5, 5.5)	0.100	2.7 (-1.3, 6.9)	0.192
Ethnicity	Non-Hispanic	2.8 (0.4, 5.4)*	0.023	1.4 (-2.0, 5.0)	0.412
	Hispanic	-2.1 (-5.9, 1.9)	0.305	-5.0 (-10.1, 0.4)	0.068
Age groups	18-64 years	1.9 (-0.4, 4.4)	0.111	-0.8 (-4.1, 2.6)	0.638
	65+ years	0.7 (-3.3, 4.8)	0.742	1.7 (-3.7, 7.4)	0.539

Table S8: Sensitivity analysis restricting to the Coastal climatic zone. Percent change in ED visits associated with heat advisory and warning issuance, by cause and demographic characteristics. Note: * p < 0.05; ** p < 0.01; *** p < 0.001.

Alert type		Advisory		Warning	
		Percent change (95% CI)	p-value	Percent change (95% CI)	p-value
**Cause-specific**					
Circulatory		0.7 (-7.8, 9.9)	0.881	-12.8 (-25.2, 1.6)	0.079
Respiratory		0.6 (-8.8, 11.1)	0.900	18.2 (0.5, 39.1)*	0.044
Mental		1.0 (-6.2, 8.8)	0.788	-7.0 (-19.1, 6.9)	0.307
Neurological		17.9 (2.9, 35.1)*	0.018	-10.0 (-30.1, 15.9)	0.414
Digestive		-4.0 (-11.4, 4.1)	0.324	-1.8 (-14.2, 12.3)	0.788
Endocrine		13.3 (-0.4, 28.7)	0.057	-9.1 (-27.7, 14.2)	0.411
Urinary		3.8 (-5.4, 13.9)	0.425	4.8 (-10.3, 22.4)	0.555
Accident		6.8 (0.9, 13.0)*	0.023	3.3 (-6.3, 13.8)	0.517
Heat-related		15.6 (-7.4, 44.3)	0.201	34.9 (-4.8, 91.0)	0.092
**All-cause**		1.5 (-0.6, 3.6)	0.157	-2.0 (-5.3, 1.5)	0.265
Sex	Male	2.1 (-0.6, 4.8)	0.121	-0.8 (-5.3, 3.9)	0.734
	Female	0.7 (-2.0, 3.6)	0.613	-3.2 (-7.9, 1.7)	0.194
Ethnicity	Non-Hispanic	1.5 (-0.8, 3.9)	0.209	-4.3 (-8.2, -0.4)*	0.033
	Hispanic	1.9 (-1.8, 5.8)	0.321	4.2 (-2.3, 11.2)	0.215
Age groups	18-64 years	2.1 (-0.1, 4.4)	0.066	-4.4 (-8.1, -0.6)*	0.023
	65+ years	-0.8 (-4.7, 3.2)	0.684	7.0 (0.0, 14.5)	0.050

Table S9: Sensitivity analysis using a distributed lag model, by lag day and cumulative effect across lags 0–3. Percent change in all-cause ED visits associated with heat advisory and warning issuance. Note: * p < 0.05; ** p < 0.01; *** p < 0.001.

Alert type	Lag	Percent change (95% CI)	p-value
Advisory	Lag 0	0.6 (-1.7, 3.0)	0.608
	Lag 1	1.2 (-1.0, 5.0)	0.147
	Lag 2	-0.9 (-3.7, 1.9)	0.512
	Lag 3	2.2 (-0.0, 4.5)	0.055
	Cumulative (lags 0–3)	4.0 (1.8, 6.3)***	<0.001
Warning	Lag 0	-1.8 (-6.1, 2.7)	0.431
	Lag 1	4.5 (-1.5, 10.8)	0.145
	Lag 2	-0.7 (-6.3, 5.3)	0.817
	Lag 3	0.0 (-4.3, 4.5)	0.995
	Cumulative (lags 0–3)	1.9 (-1.4, 5.3)	0.261

Figure S1: Monthly number of heat advisories issued by climatic zone. <u>Note:</u> Grey shading indicates the May–October warm season study period.

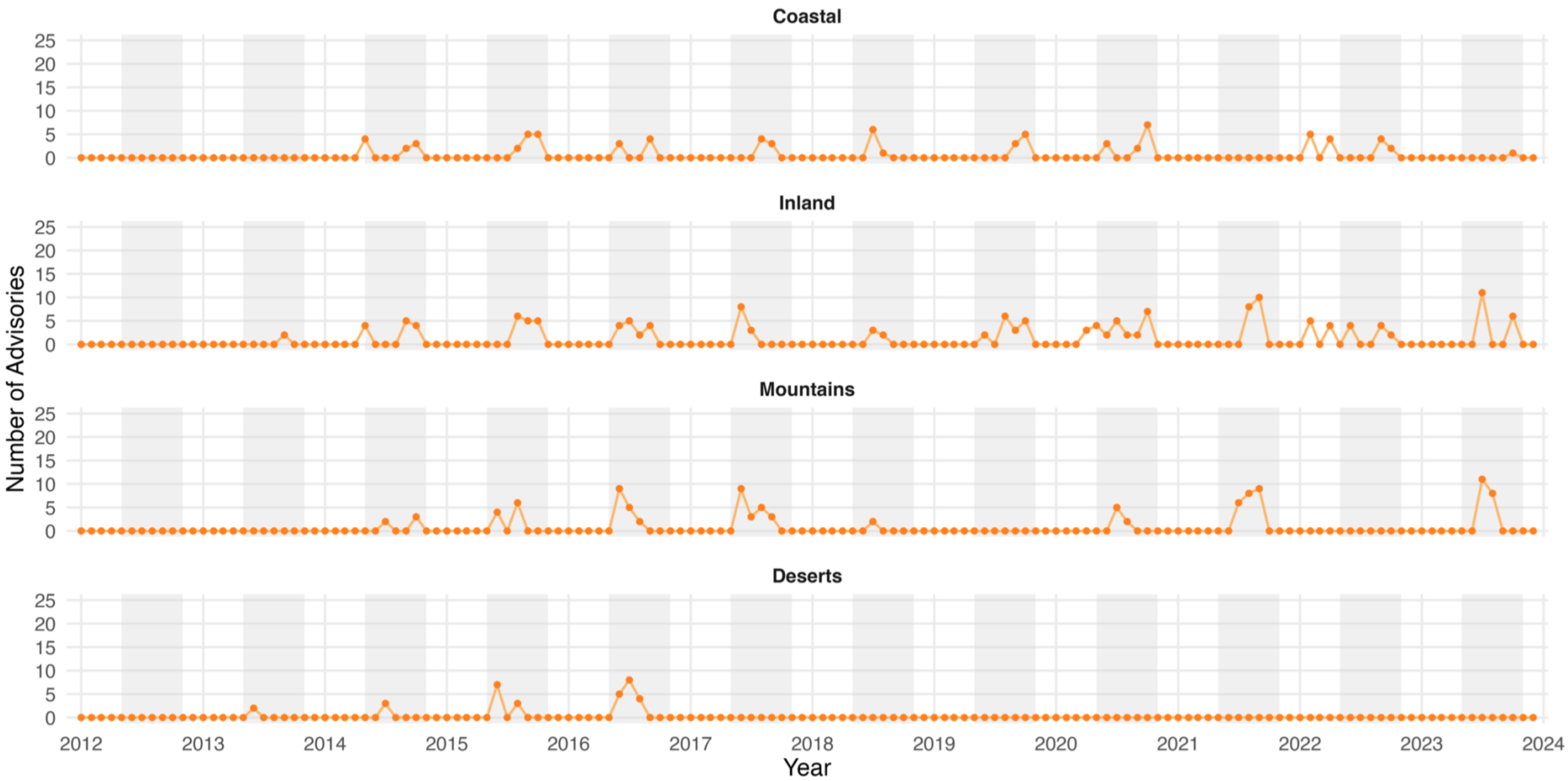

Figure S2: Monthly number of heat warnings issued by climatic zone. Note: Grey shading indicates the May–October warm season study period.

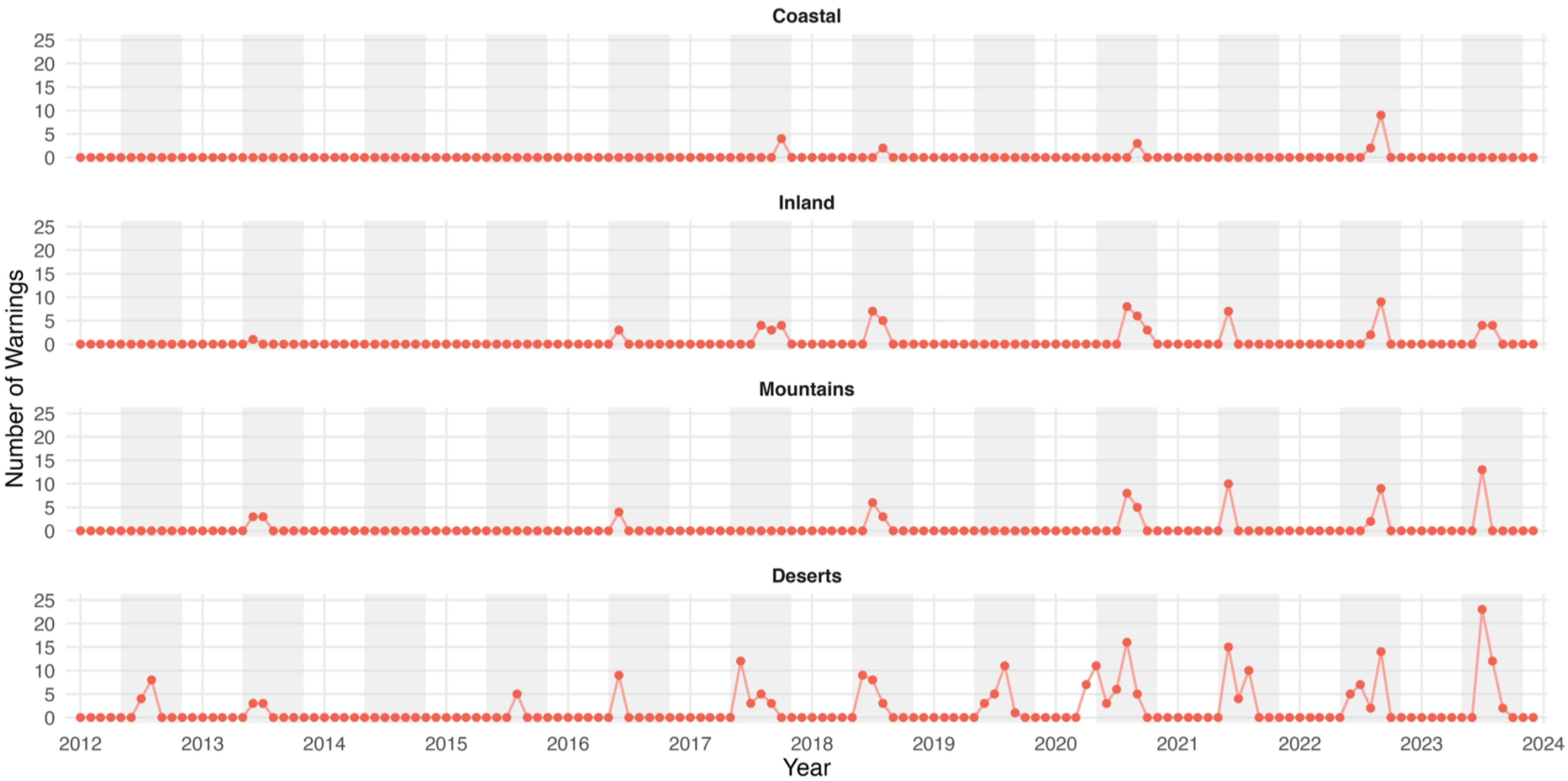

Figure S3: Pearson correlation coefficients between heat alert issuance and maximum temperature by climatic zone, 2012–2023.

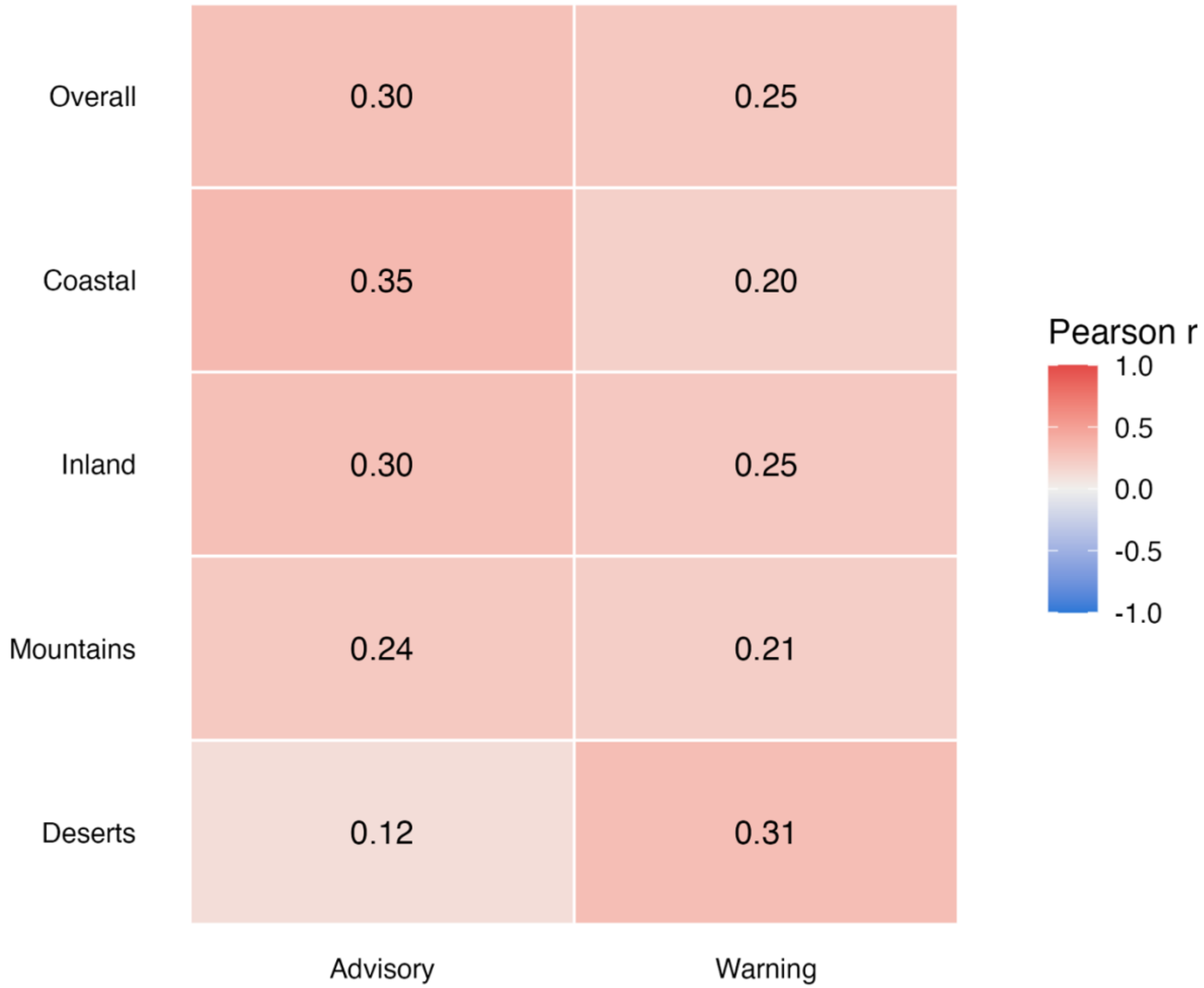

Figure S4: Geographic assignment of ZIP codes to climatic zones based on population distribution. Darker gradient shading represents a higher population share within the assigned climatic zone. Sensitivity analysis restricting to ZIP codes in which at least 80 % of the population resided within the assigned climatic zone. Note: (a) Jacobs Medical Center in La Jolla, (b) UC San Diego Medical Center in Hillcrest.

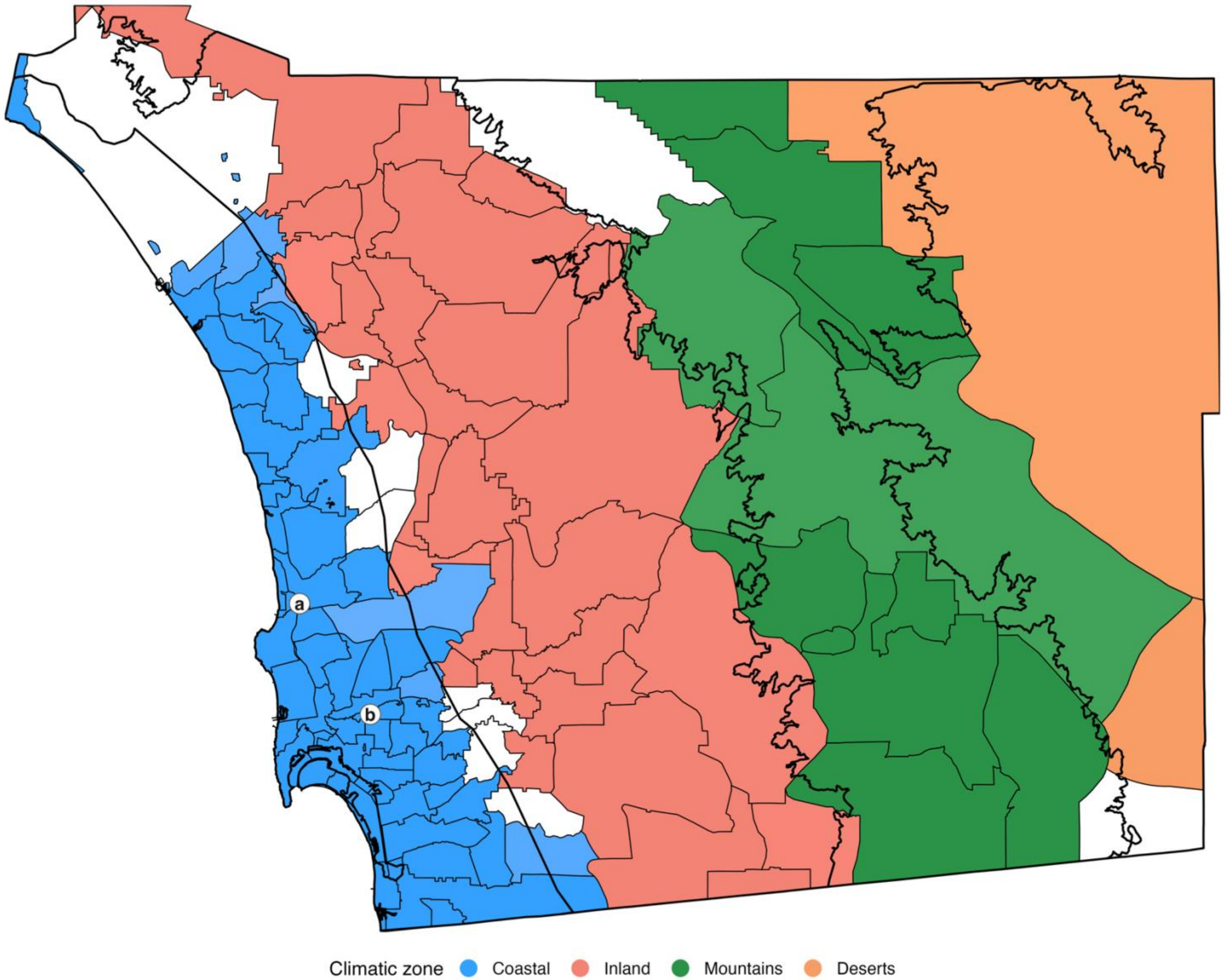

Figure S5: Sensitivity analysis restricting to ZIP codes in which at least 80% of the population resided within the assigned climatic zone. Percent change in ED visits associated with heat alert performance by demographic characteristics.

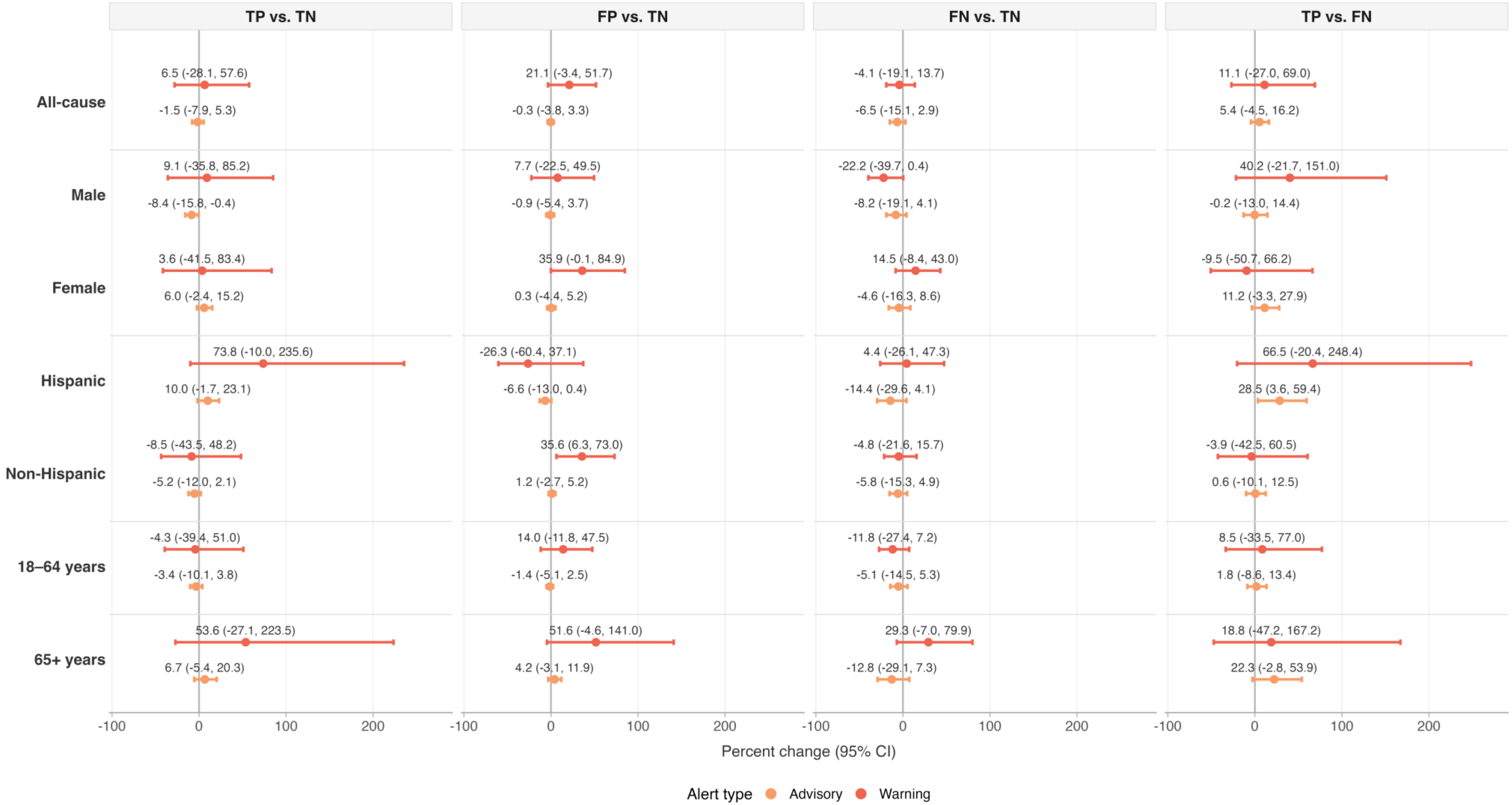

Figure S6: Sensitivity analysis excluding out-of-county residents. Percent change in ED visits associated with heat alert performance by demographic characteristics.

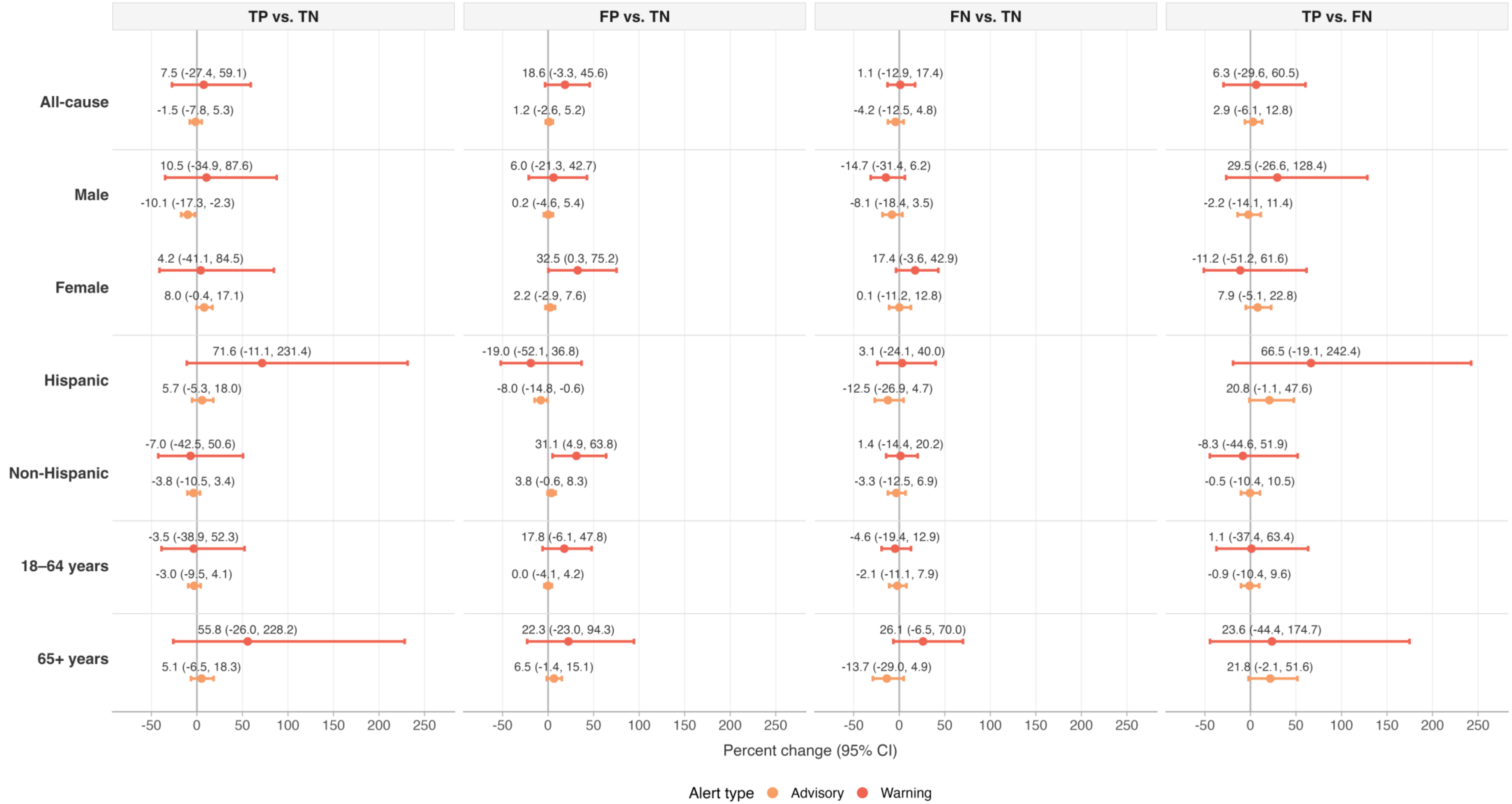

Figure S7: Sensitivity analysis incorporating a short-term temperature lag. Percent change in ED visits associated with heat alert performance by demographic characteristics.

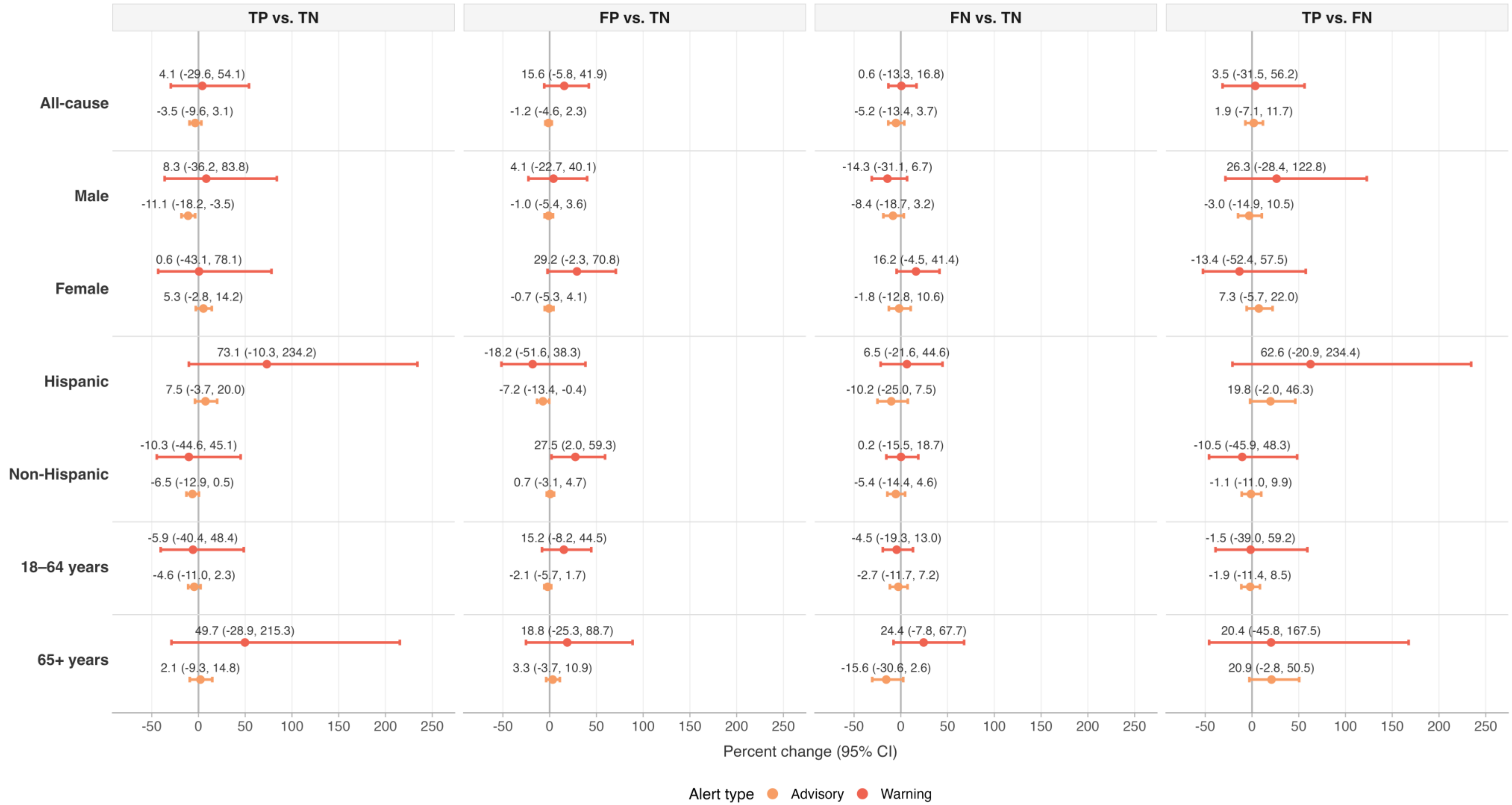

Figure S8: Sensitivity analysis controlling for relative humidity. Percent change in ED visits associated with heat alert performance by demographic characteristics.

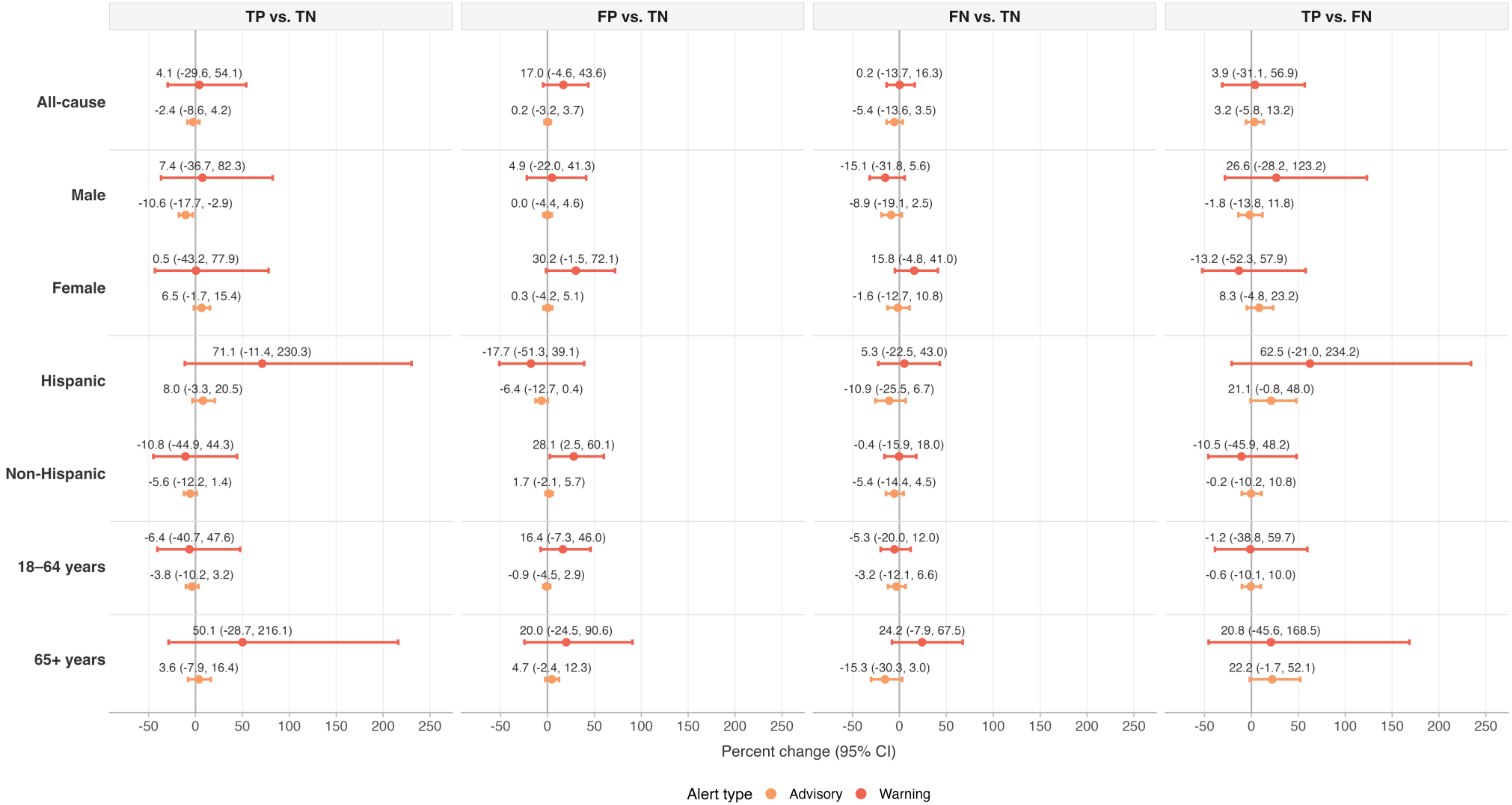

Figure S9: Sensitivity analysis restricting to the Coastal climatic zone. Percent change in ED visits associated with heat alert performance by demographic characteristics.

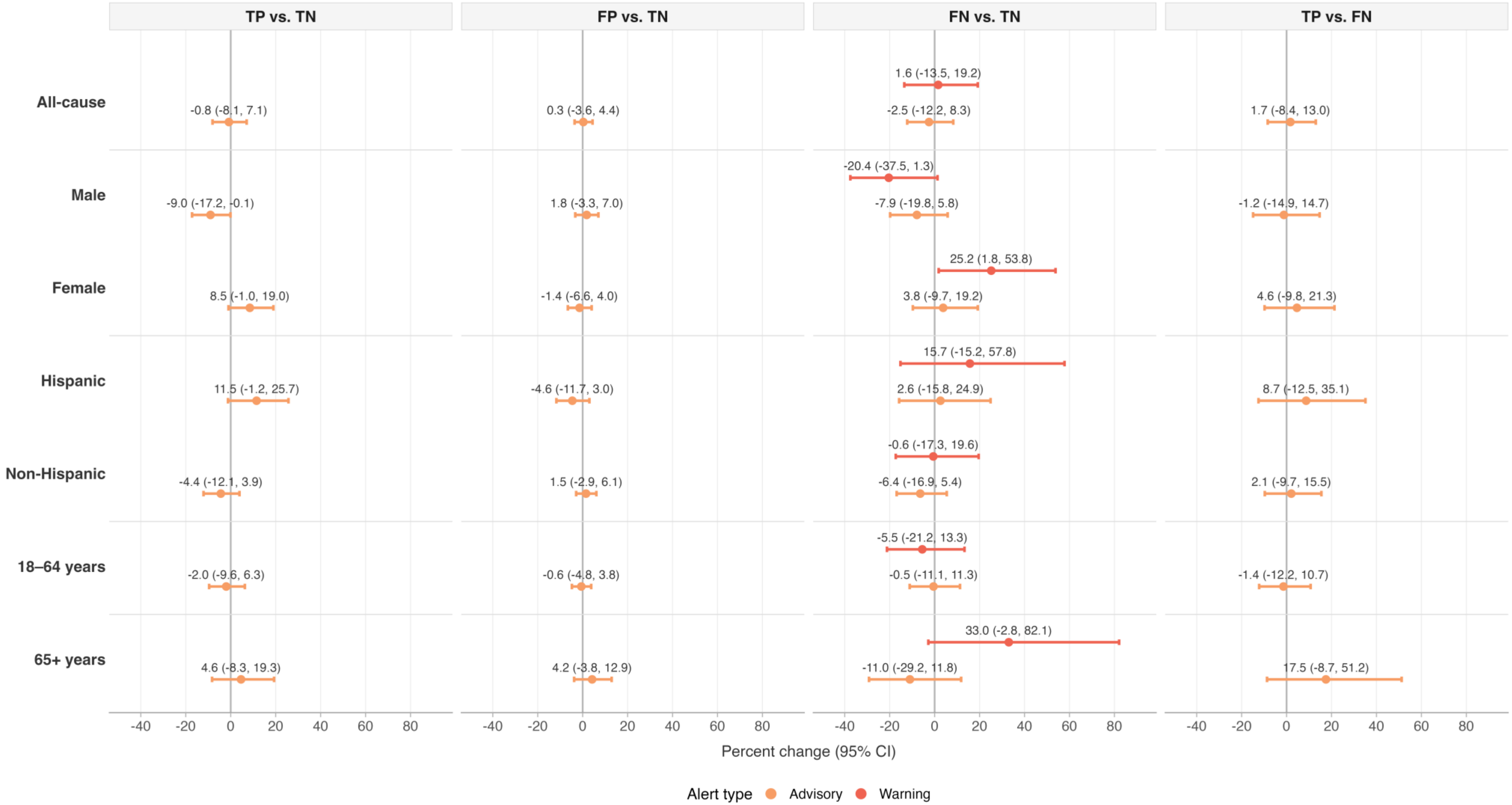

Supplement: Supplementary file 1 [file 12940_2026_1325_MOESM1_ESM.pdf]
